# Vein graft failure: Pathophysiology, detection, prevention and emerging therapeutic strategies

**DOI:** 10.1016/j.pharmr.2026.100139

**Published:** 2026-05-08

**Authors:** Simon D. Brown, Sandra Sanchez-Esteban, Laura Clark, Katarina Miteva, Georgios Krilis, Rachael O. Forsythe, Stuart A. Nicklin, Samantha Carmichael, Colin Berry, Nawwar Al-Attar, David E. Newby, Andrew H. Baker

**Affiliations:** 1 BHF Centre of Research Excellence, Institute for Neuroscience and Cardiovascular Research, University of Edinburgh, Edinburgh, United Kingdom; 2 Department of Vascular Surgery, Royal Infirmary of Edinburgh, Edinburgh, United Kingdom; 3 School of Cardiovascular and Metabolic Health, College of Medical, Veterinary and Life Sciences, University of Glasgow, Glasgow, United Kingdom; 4 Research and Innovation, NHS Greater Glasgow and Clyde, Gartnavel Royal Hospital, Glasgow, United Kingdom; 5 Department of Cardiothoracic Surgery, Golden Jubilee National Hospital, Glasgow, United Kingdom; 6 Department of Pathology, Cardiovascular Research Institute Maastricht (CARIM), Maastricht University Medical Centre, Maastricht, The Netherlands

## Abstract

Coronary and peripheral artery bypass graft surgery remain cornerstones of cardiac and vascular surgery, respectively. They utilize the saphenous vein as the preferred conduit, although it suffers from poor midterm and long-term outcomes, with the grafted vein often becoming occluded. Current therapeutic approaches aim to manage complications rather than prevent graft failure directly, and despite progress, failure rates have remained unchanged in decades, meaning there remains an unmet clinical need for novel therapeutic approaches. As access to the grafted tissue is available at the time of surgery, coronary and peripheral artery bypass graft are particularly suited to perioperative therapeutic manipulation and intervention that can be delivered locally and directly to the tissue immediately before grafting. Ongoing research attempts to uncover novel genes driving bypass graft failure that can be targeted for such therapies. In addition to protein-coding genes, multiple examples of noncoding genes driving graft failure are now described, including microRNAs and long noncoding RNAs with cell type-specific roles. The increasing wealth of single cell, single nuclei and spatial transcriptomic data related to cardiovascular pathologies are revealing novel target loci that could facilitate greater specificity for target manipulation in specific pathogenic cell types. In parallel, extensive research continues to develop advanced imaging techniques that can be used to monitor the patency of grafts over time, and guide the effective design of advanced therapies. Here, we discuss the progress in these areas and highlight how harmony among these will accelerate therapeutic progress toward vein graft disease.

**Significance Statement:**

Coronary or peripheral artery bypass surgery using saphenous vein grafts are among the most commonly performed cardiovascular surgeries for advanced vascular occlusions. However, both suffer from poor long-term graft patency. The continued development of next-generation multiomics technologies and advanced therapies are illuminating new therapeutic targets that offer hope toward novel precision medicines for peripheral and coronary artery bypass graft failure.

## Graphical abstract

**Figure:**
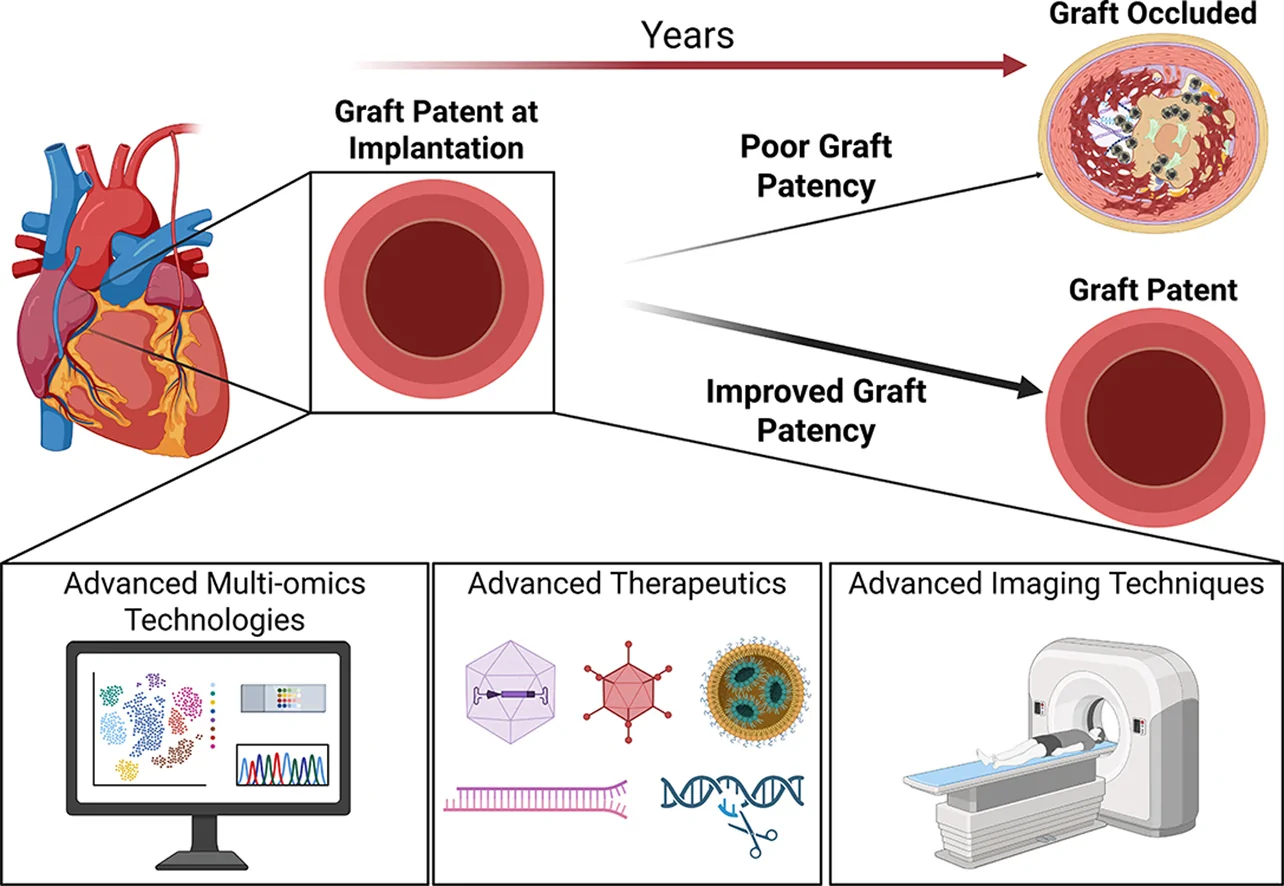

## I Venous bypass grafting: A common surgery with high failure rates

Cardiovascular disease is a major public health concern, placing an unmatched burden on international healthcare systems.^1^ With over 236 million cases reported worldwide, peripheral artery disease is becoming ever more prevalent, in part fueled by an increase in diabetes, and an expanding and ageing population.^2^^,^^3^ Chronic limb threatening ischemia is the most advanced or end stage of peripheral artery disease and is associated with substantial morbidity and cardiovascular mortality despite intervention.^4^, ^5^, ^6^ The commonest cause of death in patients with chronic limb threatening ischemia is cardiovascular disease. Indeed, ischemic heart disease remains the leading cause of cardiovascular mortality, with close to 9 million deaths being attributable to coronary events annually.^1^ Consequently, effective treatment strategies that improve both limb salvage and long term, symptom-free patient survival are paramount.

**Fig. 1:**
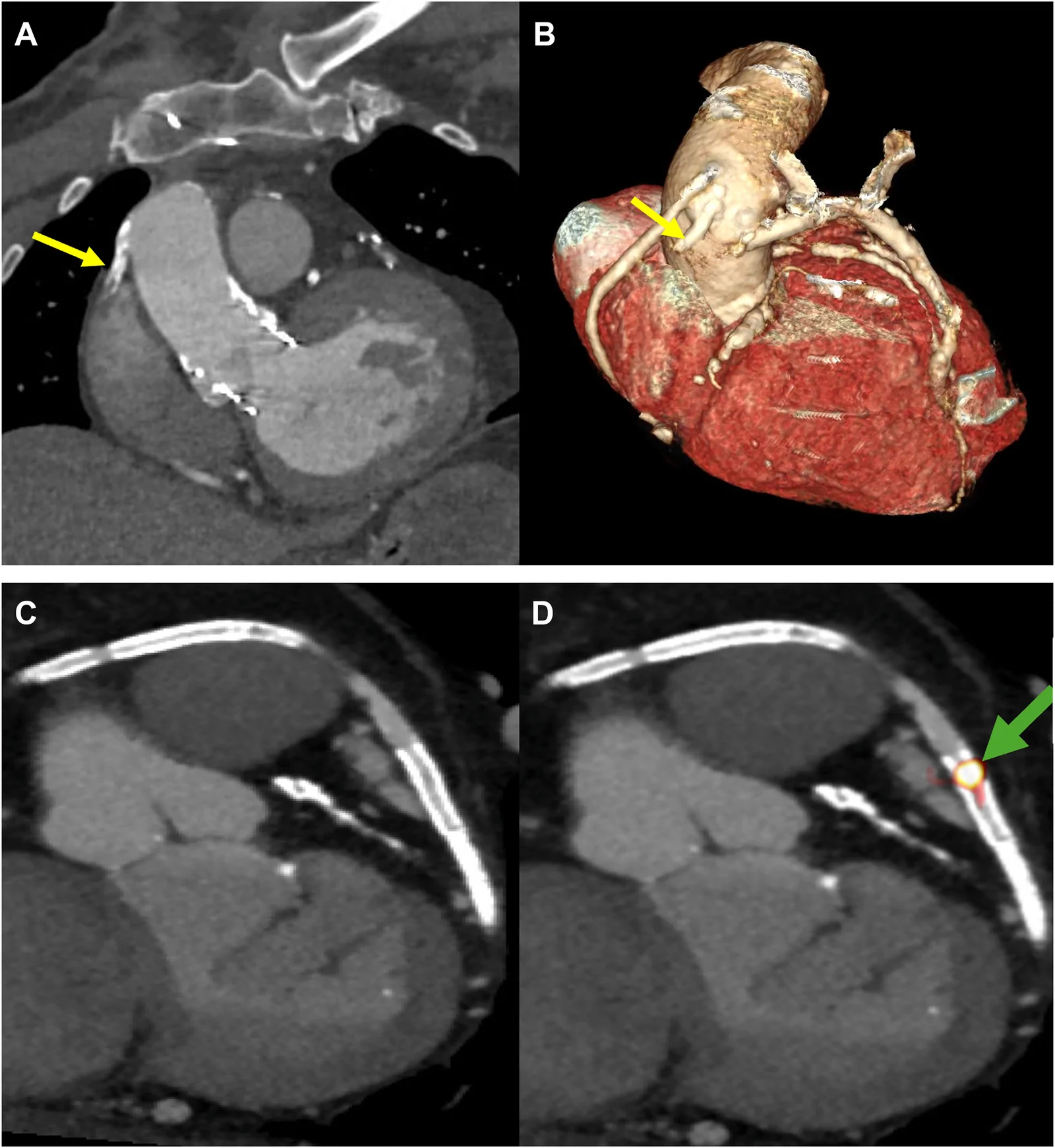
Computed tomography coronary angiography of saphenous vein CABG. (A and B) A patient presenting with stable angina with chronic saphenous vein graft failure after redo coronary artery bypass graft surgery >20 years previously (NCT06800430). Macrocalcification and occlusion of a saphenous vein graft (yellow arrow) going to the right coronary artery on computed tomography coronary angiography. This appears as truncation of the graft on 3D reconstruction, because of the absence of contrast in the distal vessel. (C and D) Adapted from Tzolos et al.^18^ A patient presenting with lateral nonspatial transcriptomics-elevation myocardial infarction and previous coronary artery bypass graft with saphenous vein graft to left circumflex artery. A stented proximal graft (prior procedure) with distal, stented culprit plaque on computed tomography coronary angiography. 18-Fluorine-GP1 uptake is visualized in the culprit lesion of the diseased saphenous vein bypass graft (green arrow)and no uptake is seen in the surrounding vessels.

Surgical revascularization forms a key management strategy to treat both coronary and peripheral arterial disease. Coronary artery bypass graft (CABG) surgery is the commonest cardiac operation, with approximately 184,000 cases performed in the United States alone annually.^7^ CABG surgery is recommended over percutaneous coronary intervention in the presence of stable left main stem, triple vessel disease, or proximal left anterior descending artery stenosis.^8^, ^9^, ^10^ During CABG surgery, autologous arterial or venous conduits are used to bypass diseased segments of the coronary tree. Arterial conduits commonly include the internal mammary and radial arteries, whereas venous conduits are taken from lengths of the great saphenous vein. Unfortunately, rates of saphenous vein graft (SVG) patency are poor, with just 50%–60% of grafts remaining open beyond 10 years postoperatively; a much lower rate than the 85%–95% patency achieved by internal mammary grafts over the same duration.^11^ Early SVG failure may be silent, with 5%–10% of patients demonstrating asymptomatic vein graft disease before discharge from hospital.^12^, ^13^, ^14^ Conversely, chronic vein graft disease may present as recurrence of ischemic symptoms, aggravated by the accelerated native coronary artery disease progression seen in patients with CABG.^15^, ^16^, ^17^ In addition to CABG and PABG, venous conduits are used across many other surgical procedures (Table 1) in a wide variety of pathologies, further underscoring the clinical importance of developing novel therapeutic approaches to prevent graft occlusion. Peri-operative angiography is performed when there is evidence of clinically significant hemodynamic compromise, or persistent ischemic changes on electrocardiography.^9^ Computed tomography coronary angiography has an increasing role in the initial investigation of suspected SVG failure, because of its noninvasive nature and ability to inform subsequent invasive angiography, reducing peri-procedural complications (Fig. 1^18^; Table 2).^19^ Redo CABG surgery is seldom performed to treat graft failure, because of the higher rates of 30-day mortality and complications such as peri-operative stroke.^10^^,^^20^ However, percutaneous coronary intervention of SVG disease carries its own challenges with increased instances of no-reflow phenomenon, in-stent thrombosis and target vessel perforation.^21^^,^^22^

**Table 1:** Major surgical procedures using saphenous vein

Surgical Procedure (Using Saphenous Vein)	Underlying Pathology/Indication	Common Complications
Coronary artery bypass grafting	Atherosclerotic coronary artery disease causing myocardial ischemia or infarction	Graft thrombosis or occlusion, intimal hyperplasia, accelerated graft atherosclerosis, myocardial infarction, leg wound infection
Femoral–popliteal (Fem–Pop) bypass	Peripheral arterial disease with femoral or popliteal artery occlusion	Graft failure, limb ischemia, graft infection, anastomotic pseudoaneurysm, hematoma
Femoral–tibial/distal bypass	Critical limb ischemia due to advanced infrapopliteal peripheral arterial disease	Early graft thrombosis, poor long-term patency, limb loss, wound breakdown, infection
Ilio–femoral bypass (selected cases, eg, after graft infection)	Severe aortoiliac occlusive disease – usually as a secondary procedure, for example, graft infection	Graft occlusion, bleeding, infection, embolization, anastomotic aneurysm
Carotid–subclavian or carotid–carotid bypass	Proximal subclavian or carotid artery occlusion (as an alternative to prosthetic or secondary procedure)	Stroke, transient ischemic attack, cranial nerve injury, graft thrombosis
Renal artery bypass	Renal artery stenosis causing hypertension or renal dysfunction	Graft stenosis or occlusion, renal infarction, worsening renal function, hypertension recurrence
Mesenteric/visceral artery bypass	Chronic or acute mesenteric ischemia	Bowel ischemia or infarction, graft thrombosis, sepsis, high perioperative mortality
Peripheral arterial reconstruction after trauma	Acute arterial injury with segmental vessel loss	Thrombosis, hemorrhage, infection, compartment syndrome, limb ischemia
Redo/infected prosthetic graft replacement	Prosthetic graft infection or failure	Recurrent infection, graft failure, bleeding, sepsis
Congenital or acquired vascular/cardiac repairs	Structural vascular defects requiring interposition grafts	Graft degeneration, thrombosis, mismatch leading to turbulence or stenosis
Arteriovenous fistula creation (vein to artery anastomosis)	Lower limb vascular access for dialysis in late-stage kidney disease with unsuitable or failed upper limb access	Graft occlusion, bleeding, infection, embolization, anastomotic aneurysm
Arteriovenous fistula salvage	Dialysis access salvage (interposition grafts)	Graft occlusion, bleeding, infection, embolization, anastomotic aneurysm
Hepatic vein reconstruction	Complex hepatopancreatobiliary surgery and liver transplant	Thrombosis, stenosis, infection, steal syndrome

Surgical procedures for which saphenous vein is commonly used as graft tissue, the underlying pathology for which surgical intervention is required, and the most common complications arising after surgery.

**Table 2:** Imaging modalities for vein graft disease

Modality	Structural or Biological	Radiotracer	Target	Process
Computed tomography angiography	Structural	NA	Vessel lumen and wall	Detection of stenosis, occlusion, or aneurysm
Optical coherence tomography	Structural	NA	Vessel wall layers; endothelium	Detection of thrombus or intimal hyperplasia
Intravascular ultrasound	Structural	NA	Vessel wall layers	Characterization of plaque burden and graft dimensions
Duplex ultrasonography	Structural	NA	Blood flow velocity and vessel wall	Assessment of graft patency and flow hemodynamics
PET	Biological activity	GP1	Glycoprotein IIIa/IIb receptors	Thrombus formation – activated platelets
PET	Biological activity	Fluciclatide	*α*v*β*3 integrin	Neoangiogenesis
PET	Biological activity	3′-deoxy-3′-F-fluorothymidine	Thymidine analog	Cell proliferation
PET	Biological activity	Fibroblast activation protein inhibitor	Fibroblast activation protein	Fibrosis – activated fibroblasts
PET	Biological activity	Fluorodeoxyglucose	Glucose analog	Macrophage metabolism – inflammation
PET	Biological activity	DOTA-0-Tyr3-octreotate (DOTATATE)	Somatostatin type II receptors	Vascular inflammation – M1 macrophages

Major imaging modalities that can be performed to study graft structure or monitor biological activity, associated radiotracers, their targets and the biological process for which they are most applicable.

Infrainguinal arterial bypass surgery is indicated for chronic limb threatening ischemia with long segment complex disease, where there is a single segment of SVG available as a conduit.^23^ Single segment SVG is the preferred conduit used in peripheral arterial bypass surgery, but as in CABG, remains prone to restenosis, with failure rates reported between 10% and 50% at intermediate follow-up.^6^^,^^23^, ^24^, ^25^, ^26^ Owing to such a high prevalence of SVG disease, international guidelines recommend routine outpatient surveillance for 2 years after open bypass surgery and the use of duplex ultrasound sonography when graft failure is clinically suspected.^23^^,^^27^ Advanced imaging with computed tomography, magnetic resonance, or digital subtraction angiography may then be used to characterize patent anatomy and plan for open or endovascular reintervention.^26^ The high recurrence and clinical impact of SVG disease underscores the pressing clinical need to develop preventative measures to combat SVG failure.

## II Mechanisms of vein graft failure

**Fig. 2:**
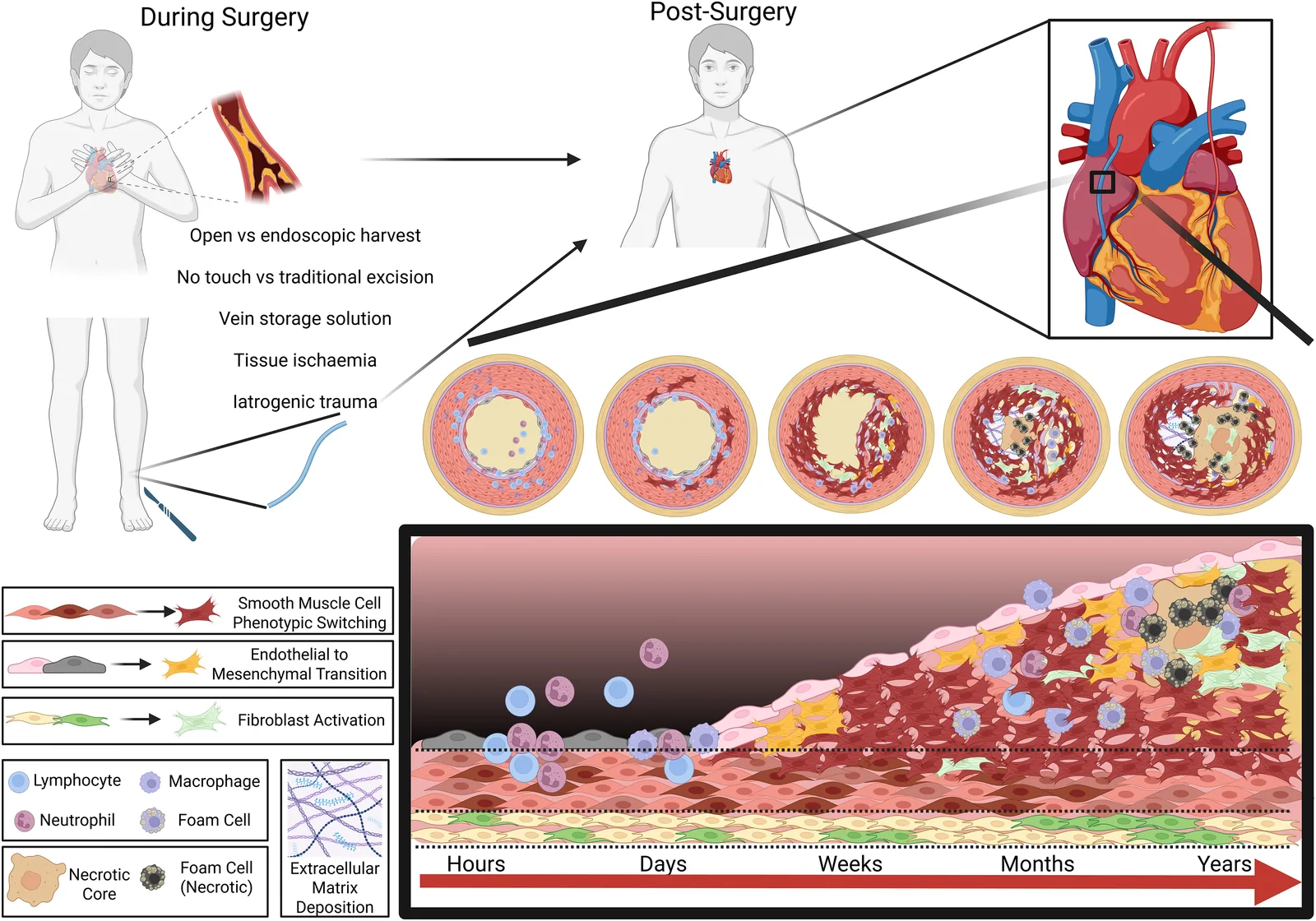
Molecular pathways leading to vein graft failure. Vein graft failure is a complex, multifactorial process, which is influenced both by decisions made before surgery and after grafting to the coronary circulation. Decisions including which tissue to use for grafting (arterial vs venous), the method of harvesting (open vs endoscopic, no touch vs traditional), the duration and composition of the storage solution, and the degree of iatrogenic damage to the tissue influence whether adaptive or maladaptive remodeling will occur, before the grafting process. Once grafted onto the heart, damage to the endothelial cell layer as a result of tissue handling, and exacerbated by ischemia-reperfusion injury, results in a proinflammatory and prothrombotic response in the vein mediated by erythrocytes, lymphocytes, neutrophils, and macrophages. The release of growth factors and cytokines from these cells as part of this response impacts the behavior of many cell types in the vessel. These include inducing endothelial-to-mesenchymal transition, phenotypic switching of vascular smooth muscle cells, and inducing fibroblast activation. Each of these processes result in widespread changes to the tissue environment that are established as proadaptive remodeling responses, however, if these processes remain unabated, they can lead to maladaptive remodeling and vein graft failure. Maladaptive remodeling can lead to an environment where an accelerated atherosclerosis-like process occurs, accompanied by the formation of necrotic cores composed of apoptotic cells and cholesterol depositions, and atherosclerotic plaques.

The mechanisms underlying CABG and PABG failure are broadly similar; therefore, although our focus is primarily centered on coronary grafts, similar processes underpin PABG failure. Vein graft failure is broadly characterized into early (<1-month postsurgery), subacute (1–12 months), and late (>12 months postsurgery).^28^ Early graft failure mostly occurs due to thrombosis in the graft, whereas subacute and late graft failure occur due to neointimal hyperplasia exacerbated by excessive smooth muscle cell proliferation and migration, and accelerated atherosclerosis, respectively. Patient characteristics including being born as a biological female, increasing age, smoking history, and diabetes mellitus are known risk factors for vein graft failure.^29^^,^^30^ However, vein graft failure begins from the moment the grafted tissue is harvested, where the tissue experiences iatrogenic trauma and a period of ischemia. Decisions made by surgeons before conduit harvest, including the method of harvest, manual handling of the graft tissue, and the graft storage solution, influence the remodeling process once the conduit is grafted into the arterial circulation. Grafting into the arterial circulation and the subsequent restoration of oxygenated blood flow through the graft further causes reperfusion injury. This combination of physical trauma and ischemia-reperfusion injury triggers a wealth of signaling cascades in the graft that collectively act to alter the phenotype of multiple cell types. Coupled with the sudden exposure to increased blood pressure, shear stress, and circumferential stress, this cocktail of effectors necessitates remodeling of the grafted tissue. Furthermore, clinical evidence suggests that the graft’s target location and quality of distal run-off impact on the patency of SVGs, with grafts to the left anterior descending artery presenting with higher patency rates relative to grafts to the right coronary artery.^31^ In this section, we will detail the specific roles of surgical processes and cell types, delineate how they contribute to vein graft disease progression, and discuss key molecular targets whose expression and function can be modulated to create novel advanced therapies. An overview of the mechanisms leading to vein graft failure are given in Fig. 2.

### A Vein graft harvesting and tissue handling

#### 1 Open versus endoscopic vein harvest

SVG harvesting is performed by 2 methods. Traditionally, “open” harvesting is performed, during which an incision is made in the patient’s leg over the course of the vein, thus exposing the vein and allowing direct visual assessment. Although open graft harvesting is commonly performed, it is associated with an increased risk of complications including edema, hematoma, wound infection, cellulitis, and wound dehiscence.^32^, ^33^, ^34^, ^35^ Alternatively, minimally invasive endoscopic harvesting can be performed, which mitigates the long incision required for open harvesting, and is reported to produce less discomfort in patients.^36^ Endoscopic harvesting is performed using a small incision at the knee, from which the vein can be visualized using an endoscope and harvested. Endoscopic harvesting is associated with reduced risk of infection, and leg-wound complications compared with open harvesting.^37^, ^38^, ^39^, ^40^ Several studies have assessed both the short- and long-term patency rates of SVGs harvested by endoscopic approach.^39^^,^^41^, ^42^, ^43^, ^44^ Yun et al^39^ performed a randomized trial in which 200 patients were assigned to have CABG surgery after either open or endoscopic vein harvesting and assessed graft patency at 6 months postsurgery. The authors noted that leg wound complications were reduced when endoscopic vein harvesting was performed and noted that the patency rate at 6 months was not different between harvesting techniques, suggesting a preference for endoscopic harvesting. Indeed, these conclusions were echoed by the publication of a consensus statement,^36^ which stated that endoscopic harvesting should be the standard of care for patients who require SVGs for coronary revascularization. However, further studies assessing long-term graft patency after endoscopic vein harvest challenged this narrative. Lopes et al^41^ assessed the rates of graft failure from patients that had undergone CABG with either open or endoscopic vein harvesting. The authors concluded that higher rates of graft failure were observed at 12 to 18 months postsurgery in patients that undergone endoscopic vein harvesting. Similarly, Andreasen et al^45^ assessed graft failure rates after a follow-up period of 6 years and asserted that vein graft failure was significantly higher in the endoscopic vein harvest group compared with the open vein harvest group. Finally, the ROOBY trial^46^ reported that endoscopic vein harvest was associated with lower 1-year SVG patency and higher 1-year revascularization rates. More recent systematic reviews and meta-analyses of multiple studies further supported these conclusions but also note a greater risk of conduit damage during endoscopic harvesting.^42^^,^^47^ Therefore, even before any surgery, the decision of which harvesting technique to use can have a major impact on the progression of graft failure.

#### 2 No-touch technique and vein preservation protocols

Saphenous vein harvesting frequently involves extensive denudation of the tissue and repeated distension to ensure there are no leaks before grafting in the heart, and to alleviate the risk of spasm occurring after surgery. These processes, although important to ensure structural integrity of the vein, have been identified as key contributors to tissue damage, particularly to the endothelial cell layer.^48^^,^^49^ To combat the extensive tissue damage reported as a result of traditional harvesting techniques, the “no-touch” technique was developed.^50^ Whereas traditional vein excision involves exposing the vein and stripping the majority of the surrounding tissue, including the perivascular fat, the “no-touch” technique maintains a cushion of perivascular fat and adventitial nerves surrounding the saphenous vein. Several small-scale, single-center clinical trials reported that harvesting using the “no-touch” technique resulted in greater graft patency compared with traditional harvest techniques.^51^, ^52^, ^53^, ^54^, ^55^ However, in larger, multicenter clinical trials, the “no-touch” technique was not associated with improved patency of SVGs after CABG at 1 year after surgery^56^ but was associated with a greater increase of infection at the site of harvest.^56^ To date, the benefit of the “no-touch” technique remains controversial with several recent studies reporting no superior outcomes using the “no-touch” technique,^57^, ^58^, ^59^ whereas another study reported better graft patency.^60^ Importantly, the SWEDEGRAFT trial also reported more leg wound complications with “no-touch” technique at 3 months.^57^

Once harvested, the saphenous vein remains outside the body for a period of time before grafting onto the heart. In an effort to protect against ischemic-reperfusion injury and promote endothelial integrity, the impact of the storage solution on graft patency has been assessed.^61^ DuraGraft is a vascular conduit preservation solution designed to preserve the endothelium of the saphenous vein.^62^ It is primarily composed of a physiological salt solution containing glutathione and L-ascorbic acid, antioxidants, and arginine. Arginine is reported to act as a substrate for nitric oxide (NO) synthase in endothelial cells, which the inventors claim offers systematic protection of the endothelium against ischemic injury during storage. An ex vivo study performed by Thatte et al^62^ reported that DuraGraft solution was superior to both saline and blood in their ability to protect the endothelium of human saphenous vein. A randomized, within-patient, phase 1 clinical trial (NCT02272582/NCT02774824) has been reported to be completed.^63^ However, no data have emerged from these studies comparing DuraGraft to the current standard of care within patients undergoing saphenous vein CABG.

Cavallari et al^64^ investigated how the composition of the storage solution impacts neointima formation. The authors compared storage of canine jugular and femoral veins for 45 minutes in either autologous whole blood, 0.9% normal saline solution, and University of Wisconsin solution before grafting. The study reported increased intimal thickness in veins that had been stored in autologous whole blood or 0.9% normal saline solution relative to storage in University of Wisconsin solution. A recent consensus statement from the European Society of Cardiology Working Group on Cardiovascular Surgery and the European Association for Cardio-Thoracic Surgery Coronary Task Force^65^ recommends that buffered solutions should be used in place of autologous blood or saline solutions. This conclusion is supported by multiple studies demonstrating improved preservation of endothelial structural integrity and function compared with both autologous blood and saline.^66^, ^67^, ^68^ The consensus statement also does not currently support the use of endothelial damage inhibitors as no large-scale comparative studies have been performed with regard to graft patency or clinical outcomes.

Collectively, this body of work demonstrates that even before surgery beginning, multiple decisions (including harvesting technique, skeletonizing the vessel, and short-term storage solution) can have consequences on graft patency, and thus the outcome of patients.

### B Ischemia-reperfusion injury

During bypass surgery, the grafted conduit undergoes significant physical trauma including repeated distension involving pressurizing the vessel to check for leakage. After excision, the absence of blood, and therefore of oxygen, glucose, and other important metabolic compounds, means that the vein undergoes a period of ischemia, followed by reperfusion injury after grafting when blood flow is restored. The combined action of these conditions results in extensive damage to the vessel endothelium. Ischemia results in reduced oxygen availability in the tissue, during which the cells exist under hypoxic conditions. Hypoxia has been shown to directly contribute to increased endothelial cell damage in vitro^69^ and ex vivo.^70^ Hypoxia results in the increased expression of multiple genes associated with reactive oxygen species production, including NADPH oxidases, xanthine oxidases, cyclooxygenase, NO synthase, lipoxygenase, as well as leading to increased production of mitochondrial reactive oxygen species.^71^ Collectively, the increased production of reactive oxygen species such as superoxide (O_2_^−^) and hydrogen peroxide (H_2_O_2_)^72^ promote thrombus formation and leads to further pathogenic changes to endothelial cells, smooth muscle cells and extracellular matrix production, each of which are discussed below.

Once blood flow has been restored after engraftment, the sudden influx of oxygenated blood through the grafted tissue can cause further damage to the graft.^70^^,^^72^ During ischemia, mitochondrial anaerobic glycolysis causes glycogen to be broken down into 2 molecules of ATP and lactic acid. This continual process results in a lowering of the intracellular pH, which acts as a negative feedback loop to prevent further ATP production. ATP then becomes sequentially cleaved to produced ADP, AMP, and IMP, which are then further broken down into adenosine, inosine, hypoxanthine, and xanthine. Once the tissue is grafted and blood flow is restored, the influx of oxygen in the blood catalyzes xanthine oxidase to degrade hypoxanthine to uric acid. This liberates the highly reactive superoxide anion (O_2_^−^), which is subsequently converted to hydrogen peroxide (H_2_O_2_) and the hydroxyl radical (OH^•^).^73^ This results in the peroxidation of lipid structures, which trigger the production and release of proinflammatory eicosanoids, disruption of cell permeability and ultimately cell death.^74^

### C Early graft failure: De-endothelialization and graft thrombosis

During vein harvest, the endothelial cell layer undergoes trauma. The endothelium is a semipermeable cellular barrier between circulating blood and the vasculature that is fundamental for vascular homeostasis. Endothelial cells help to regulate vascular permeability and tone through the regulated production of vasodilatory (NO, prostacyclin [PGI2], and endothelium-derived hyperpolarizing factor) and vasoconstrictive signaling factors (endothelin-1, thromboxane A2, and angiotensin II).^75^ Damage to the endothelial cells results in an imbalance in these signaling cascades, most prominently a decrease in NO levels. Through its action as a vasodilatory signaling molecule, NO is essential not only in endothelial cell homeostasis, but also in the regulation of constrained vascular smooth muscle cell proliferation,^76^ reduced platelet aggregation, and reduced leukocyte adhesion.^77^ Reduced NO, consequently, leads to reduced vasodilation and an acceleration of the processes leading to vein graft failure.^78^, ^79^, ^80^

Thrombosis is the predominant mechanism of early graft failure (failure within the first month of surgery).^81^^,^^82^ Endothelial cell damage, and subsequent loss, expose the underlying extracellular matrix to platelets in the blood. The extracellular matrix contains high levels of the adhesive protein’s fibrinogen, von Willebrand factor, and the fibrillar collagen types I and III.^83^^,^^84^ Platelets bind directly to fibrinogen and von Willebrand factor already present in the extracellular matrix.^84^, ^85^, ^86^ Collagens present in the extracellular matrix are potent platelet agonists that contribute to platelet aggregation.^87^ Platelet aggregation contributes to remodeling of the extracellular matrix, including a reduction in the elastin/collagen ratio (a major determinant of vascular stiffness) and an increase in fibrillin-1.^88^

Iatrogenic damage to the endothelium can also cause endothelial cells to alter their phenotype to an “activated” state. Activated endothelial cells present with increased production of cell adhesion proteins such as intercellular adhesion molecule-1, vascular cell adhesion protein-1, as well as selectins, thrombomodulin, cytokines, and growth factors,^89^, ^90^, ^91^, ^92^, ^93^ and reduced production of the vasodilators and antithrombotic factors prostacyclin, NO, and endothelium-derived hyperpolarizing factor.^94^, ^95^, ^96^ This signaling cascade promotes a proinflammatory and prothrombotic response, including increased leukocyte adhesion and transmigration through the damaged endothelium,^97^ and increased platelet activation.^11^^,^^96^^,^^98^^,^^99^ Collectively, these processes assimilate to promote thrombus formation leading to early graft failure. Current clinical practice recommends the use of antiplatelet and antithrombotic drugs such as aspirin alone,^100^ or dual antiplatelet therapy with Clopidogrel plus aspirin.^65^^,^^101^ Aspirin administration after CABG is routinely performed based on the results of several randomized controlled trials demonstrating the benefit of aspirin for the prevention of SVG occlusion at both early^102^ and at 1-year postsurgery.^103^

Graft endothelial quality may be assessed invasively both in vivo and ex vivo using optical coherence tomography, where a near-infrared light is used to produce cross sectional images of the vessel wall.^104^^,^^105^ A single center randomized control trial is currently ongoing, to investigate whether optical coherence tomography guided conduit assessment before grafting can reduce the rates of SVG failure (NCT05129228).

Positron emission tomography (PET) is a noninvasive imaging technique, which may also be used to assess graft thrombosis, through the targeted imaging of activated platelets. The radiotracer 18-fluorine-GP1 binds to glycoprotein IIb/IIIa receptors on activated platelets and has been used to identify platelet activation within the culprit lesion of a diseased SVG (Table 2).^18^

### D Re-endothelialization

Although the mechanisms underlying endothelial cell damage and activation are established, the process of endothelial cell repair and regeneration remain comparatively less-well understood.^106^ After injury, 2 important processes must occur through endothelial repair and regeneration: complete restoration of the endothelial cell monolayer, and the reestablishment of endothelial cell junctions and barrier function.^107^ Together, these constitute the first steps toward complete neovascularization, with correct flux between the blood and the vasculature re-established and the restoration of semipermeability of endothelium. Endothelial cell regeneration is believed to occur from resident endothelial cell proliferation and migration, but has also been suggested to occur via the recruitment of a heterogeneous population of endothelial progenitor cells (EPCs) that can differentiate into endothelial cells.^108^^,^^109^ Since the discovery of this endothelial progenitor stem cell population,^110^ significant research has been undertaken to establish the contribution of these cells to angiogenesis and vascular pathology.^111^, ^112^, ^113^, ^114^, ^115^ However, despite decades of research, EPCs studies remain unclear and controversial, with everything from their origin^111^^,^^116^ and identification,^117^ as well as their role in the pathophysiology of cardiovascular disease a source of debate, with research findings frequently reporting contradictory results.^118^ Kawamoto et al^119^^,^^120^ reported that intramyocardial transplantation of unmodified EPCs could enhance neovascularization to the ischemic myocardium of pigs^119^ and rats,^120^ suggesting the potential for a novel cell therapy toward myocardial ischemia. Similar studies in animal myocardial infarction models have reported improved blood flow and cardiac function.^121^^,^^122^ Further studies have shown that adenoviral vector–transduced EPCs, expressing vascular endothelial growth factor (VEGF) or hypoxia inducible factor 1*α*, enhanced neovascularization in murine ischemia models.^123^^,^^124^ Clinical studies have demonstrated that patients presenting with acute myocardial infarction have double the number of EPCs compared with patients with stable coronary artery disease,^125^^,^^126^ suggesting a link between myocardial ischemia and EPC recruitment. Despite these promising links, EPCs have also been reported to accelerate atherosclerosis.^127^^,^^128^

The most commonly used cellular markers for EPCs are CD34, VEGF receptor-2, and CD133.^110^^,^^129^ However, these markers are not unique, and most are also expressed in other hematopoietic cells and mature endothelial cells. VEGF receptor-2 and CD34 are expressed in both hematopoietic stem and progenitor cells. Specifically, CD34 is expressed by microvascular endothelial cells, hematopoietic progenitors and hematopoietic stem cells. Case et al^130^ performed hematopoietic and endothelial cell clonogenic assays that demonstrated that cells isolated from bone marrow and peripheral blood using these classical EPC markers were not true EPCs, with the majority of cells classed as hematopoietic progenitor cells, which express CD45, a recognized hematopoietic cell surface marker. Among the total population of cells, the authors noted a small subpopulation of CD45^−^ cells that do not exhibit hematopoietic activity and could be genuine EPCs.

These observations cloud our understanding of the precise role of EPCs in cardiovascular pathology and illustrate the complexity there remains to unravel before we are to realize the therapeutic potential of EPC cell therapies.

### E Molecular mechanisms leading to late graft failure and neointimal hyperplasia

#### 1 Endothelial-to-mesenchymal transition

Damage to the endothelial cell layer, the subsequent inflammatory and cytokine response, and the increase in reactive oxygen species, has been shown to trigger endothelial-to-mesenchymal transition (EndMT).^131^, ^132^, ^133^ EndMT is the process by which endothelial cells lose the expression of classical endothelial cell-specific markers such as CD31, VE-cadherin, and endoglin and alter their morphology toward a mesenchymal cell-like phenotype, coupled with an increase of myogenic markers such as *ACTA2* (α smooth muscle cell actin, αSMA) and *TAGLN* (smooth muscle protein 22 alpha, SM22α).^133^ A wealth of research has suggested that EndMT contributes to the development of several cardiovascular diseases including atherosclerosis,^134^, ^135^, ^136^, ^137^, ^138^, ^139^ CABG failure,^131^ valvular disease,^140^, ^141^, ^142^, ^143^, ^144^ and pulmonary arterial hypertension,^145^, ^146^, ^147^, ^148^, ^149^ whereas conflicting research suggests the precise role of EndMT in cardiac fibrosis remains unclear.^150^, ^151^, ^152^, ^153^ At the molecular level, EndMT is initiated through the concerted action of several signaling molecules that act to induce the expression of transcription factors including Snail, Slug, Twist, *ZEB1*, *ZEB2*, and *SOX2*.^154^ Among the most-established canonical signaling molecules in the initiation of EndMT are the transforming growth factor (TGF)-*β* and bone morphogenetic protein family of growth factors, which act to induce the expression of these transcriptional factors through both Smad-dependent and Smad-independent pathways.^155^^,^^156^ Smad-independent pathways include acting via MAP kinase, Rho-like GTPase, ERK1/2, or phosphatidylinositol-3-kinase/AKT.^157^ This wide family of signaling molecules are established, and indeed, indispensable in the initial response to many physiological processes including wound healing, inflammation and increased shear stress, which are hallmarks of the early adaptation tissues experience after successful engraftment during CABG surgery.^154^ Although these canonical pathways are recognized across many studies for both developmental EndMT and pathogenic EndMT, several noncanonical pathways have been detailed for developmental EndMT, including Notch and Wnt/*β*-catenin signaling, although evidence for these pathways in the development of pathological EndMT has yet to be established.^158^ Recent evidence has identified noncoding genes, including both microRNAs (miRNAs) and long noncoding RNAs (lncRNAs), which have important functions in the initiation and promotion of EndMT. LncRNAs have been suggested to possess more cell and tissue-specific expression patterns,^159^, ^160^, ^161^, ^162^, ^163^, ^164^, ^165^ suggesting they may offer benefits as molecular targets as theoretically, there could be fewer off-target effects as a result of altering their expression levels. MiRNAs are reported to efficiently bind to and downregulate the expression of many mRNA targets,^166^^,^^167^ suggesting that targeting miRNAs can be a powerful approach to alter phenotypes by targeting a single miRNA.

Ghosh et al^168^ used TGF-*β* to induce EndMT in adult mouse cardiac endothelial cells and performed microRNA arrays to identify miRNAs that are differentially expressed upon EndMT induction. Among many induced miRNAs, miR-21 and miR-27b were reported to be upregulated. Both miR-21 and miR27b have been reported to possess proangiogenic functions related to neointima formation.^169^^,^^170^ Inhibition of miR-21 was sufficient to restore both the TGF-*β*-induced repression of VE-cadherin and the induced expression of S100A4.^171^ Therefore, these noncoding genes offer hope for the development of specialized therapeutic modalities to prevent, or even reverse EndMT.^158^

Given that EndMT cells display increased proliferation and migration and secrete matrix metalloproteinases (MMPs) that act to promote neointimal hyperplasia,^172^ blocking, or even reversing EndMT, is an important therapeutic goal toward the prevention of CABG failure.

#### 2 Smooth muscle cell phenotypic switching

In adult blood vessels under healthy physiological conditions, vascular smooth muscle cell function is primarily centered around regulating vascular tone, facilitated through the expression of several contractile genes including *α*-smooth muscle actin, calponin, smooth muscle myosin heavy chain, caldesmon, and metavinculin.^173^^,^^174^ Vascular smooth muscle cells exhibit extremely low levels of proliferation under healthy physiological conditions, but upon insult, such as iatrogenic damage, rapidly dedifferentiate and change phenotype from a contractile, quiescent state to a synthetic, proliferative, and promigratory state.^174^^,^^175^ Proliferating vascular smooth muscle cells release a variety of cytokines, growth factors, and matrix-degrading proteases that trigger downstream signaling pathways, leading to an exacerbation of the proliferative response.^176^^,^^177^ These processes are part of the normal adaptive response of the vein acclimatizing to the arterial circulation but can become maladaptive if excessive proliferation occurs. Excessive proliferation of smooth muscle cells leading to neointimal hyperplasia is an important pathological feature of many vascular pathologies including primary atherosclerosis, peripheral artery bypass graft (PABG) failure, arteriovenous dialysis access, postangioplasty restenosis, and transplant vasculopathy. Blocking smooth muscle cell proliferation as a therapeutic approach toward vascular pathology using a variety of modalities has been assessed numerous times across many studies (discussed below); however, despite this, no therapeutic modality targeting smooth muscle cell proliferation or migration has reached clinical use. This is as a result, primarily, of a noncell-selective block of proliferation, meaning processes such as re-endothelialization are often impaired, leading to many off-target effects. This has led to efforts to identify vascular smooth muscle cell-specific targets, particularly noncoding RNAs, which are either not expressed in other vascular cell types, or which, in response to specific stimuli, primarily function in vascular smooth muscle cells.^178^ Rodor et al^179^ performed an unbiased miRNA screen by transfecting miRNA mimics into primary human smooth muscle cells and assessing changes to interleukin (IL)-1*α* and platelet-derived growth factor-induced proliferation. From a screen of more than 2000 miRNA mimics, the authors were able to identify 7 novel miRNAs that reproducibly block proliferation in vitro across vascular smooth muscle cells from several vascular beds, without affecting proliferation of vascular endothelial cells. The authors further demonstrated that delivery of some of these miRNAs’ ex vivo to human saphenous vein tissue could block proliferation in whole tissue, suggesting their potential for use as a novel RNA therapy.

In addition to exhibiting increased proliferation and migration, synthetic vascular smooth muscle cells also release high levels of MMPs^84^^,^^180^ that act to degrade the basement membrane and extracellular matrix, exacerbating the proliferative and promigratory capacity and accelerating neointima formation.^88^ MMP activity is balanced by an endogenous family of proteins, the tissue inhibitor of metalloproteinases (TIMPs). Overexpression of members of the TIMP family has been shown to block rat vascular smooth muscle cell proliferation in vitro via increased smooth muscle cell apoptosis.^180^ Adenoviral-mediated overexpression of *TIMP-3* has also been shown to reduce smooth muscle cell proliferation in human saphenous vein tissue^181^ and in large porcine models of vein graft failure,^181^^,^^182^ suggesting that gene therapy centered around overexpression of *TIMP-3* is a promising therapeutic approach to prevent vein graft failure.

Intravascular ultrasound may be used to quantify early graft intimal hyperplasia in vivo (Table 2).^183^^,^^184^ However, the invasive instrumentation of mature SVGs can result in significant complications, limiting the use of serial and late intravascular ultrasound imaging in randomized controlled trials. In contrast, peripheral SVGs are amenable to serial duplex ultrasound imaging, owing to the superficial anatomical location of the graft, providing valuable information about early SVG remodeling and predicting subsequent graft failure.^185^ However, as with other imaging techniques, duplex ultrasonography is unable to provide biological information on SVG disease, providing another niche for PET imaging (Table 2).

#### 3 Fibroblast activation

Fibroblasts are a highly abundant cell type, whose primary function is to maintain the production of extracellular matrix and provide structural support to surrounding cells and tissues. Fibroblasts possess a high degree of plasticity, and in response to specific stimuli can be reprogrammed into other cell types.^186^ Adventitial fibroblasts are an essential contributor to wound healing and tissue repair processes after bypass graft surgery,^187^ and have evolved to rapidly differentiate into many fibroblast subtypes in response to local stimuli, or in response to altered physiological states, or aging.^188^^,^^189^ These differentiated fibroblast subtypes influence their surrounding environment through the increased secretion of growth factors, cytokines, and chemokines and are key drivers in fibrosis, inflammation, and angiogenesis. There is, therefore, significant interest and importance in understanding the role of fibroblasts, and their subtypes, in the development of vein graft failure.

Fibroblast activation protein inhibitor (FAPI) can be labeled with fluorine-18 ([Al18F]-FAPI) or gallium-68 ([68Ga]-FAPI)^190^ to generate a positron-emitting radiotracer for the in vivo detection of activated fibroblasts (Table 2). To date, the use of FAPI PET imaging in cardiovascular disease has predominantly focused on myocardial infarction.^191^, ^192^, ^193^ However, the first noninvasive study using [^68^Ga]-FAPI-46 to explore the temporal and anatomical distribution of SVG fibrosis is currently underway (ClinicalTrials.gov, NCT06800430).

#### 4 Inflammatory responses: Positive and negative

The proinflammatory response initiates during surgical excision and continues after grafting into the coronary circulation; this is part of the natural response to trauma and an important initiator of the wound healing response. This systemic inflammatory response is orchestrated by a complex network of signaling pathways mediated through the activation of the complement cascade and the release of cytokines from neutrophils, mast cells, leukocytes, monocytes, natural killer cells, macrophages, and lymphocytes.^194^, ^195^, ^196^ During CABG surgery, damage-associated molecular patterns are released that bind to Toll-like receptors and initiate the release of cytokines such as IL-1*α*, IL-1*β*, IL-6, and tumor necrosis factor-*α* (TNF-*α*), further magnifying the inflammation response.^196^ Through their interaction with Toll-like receptors, and the IL-1, IL-6, and TNF receptors,^195^, ^196^, ^197^, ^198^ cytokines activate intracellular signaling pathways, including the mitogen-activated protein kinase, nuclear factor-*κ*B (NF-*κ*B), and Janus kinase (JAK)-signal transducer and activator of transcription (STAT) pathways.^196^^,^^198^^,^^199^ Given their key roles influencing several cellular processes that collectively lead to the pathology of cardiovascular diseases, including vein graft failure, significant research has focused on retarding the IL-6 and JAK/STAT pathways. IL-6 mediates its cellular effects via the signal transducer gp130.^200^ IL-6, after receptor binding, homodimerizes and activates gp130. Activated gp130 subsequently recruits nonreceptor tyrosine kinases such as JAKs, which in turn, phosphorylate gp130.^201^ Phosphorylated gp130 creates docking sites for the downstream signal transduction molecules, including STAT-3.^202^ These studies illuminate the elegant interplay between the IL-6 and JAK/STAT signaling pathways and emphasize how manipulation of these pathways can have significant widespread changes to many downstream networks.

JAK1/2-mediated STAT-3 expression is associated with the dedifferentiation, proliferation, and migration of vascular smooth muscle cells,^203^^,^^204^ suggesting that inhibiting this pathway could be a viable therapeutic approach to prevent vein graft failure. As JAK-STAT signaling is important in the development of many other diseases, a considerable amount of work has been dedicated to identifying selective therapeutic inhibitors of STAT family members, particularly STAT-3.^205^ These range from small molecule inhibitors, antisense oligonucleotides (ASOs) and oligodeoxynucleotide decoys, most of which focus on restricting STAT-3 phosphorylation and/or dimerization.^205^ Daniel et al^203^ demonstrated that WP1066, a small molecule that inhibits the phosphorylation of JAK2 and STAT-3, inhibited proliferation of vascular smooth muscle cells and decreased the neointimal lesion size in a mouse wire-induced injury model. In another study, the STAT-3 inhibitor apigenin was reported to increase the expression of contractile markers and block platelet-derived growth factor-induced proliferation both in vitro in smooth muscle cells and in vivo in a mouse carotid arterial injury model.^206^ In the context of neointima formation, inhibition of gp130-STAT-3 signaling suppressed balloon injury–induced STAT-3 phosphorylation, cyclin D1 expression, smooth muscle cell migration, and neointima formation in a rat balloon injury model.^207^ These studies indicate that inhibition of STAT-3 signaling attenuates the proliferative and migratory response of vascular smooth muscle cells after vascular injury and has the potential to be used to prevent vein graft failure clinically. Although there are many ongoing clinical trials exploring the potential use of STAT-3 inhibitors, clinical studies exploring these in a cardiovascular context, or specifically in a vein graft context, are currently not in progress. It is important to note that inhibition of STAT-3 signaling may present with significant off-target effects, which could be detrimental post-CABG. For example, several studies have reported that STAT-3 has functions in preventing viral infections^208^, ^209^, ^210^, ^211^ and bacterial infections,^212^ suggesting that treatment of patients during vein graft surgery with STAT-3 inhibitors may lead an increased susceptibility to postsurgical infections.

18-Fluorine-fluorodeoxyglucose is a radiolabeled glucose analog, which when used for the imaging of atherosclerosis, is thought to represent hypoxic macrophage metabolism (Table 2).^213^ Theoretically, 18-fluorine-fluorodeoxyglucose could be used to identify in vivo inflammation present in SVG disease. So far, its use has been limited to a single case report identifying inflammation secondary to infection of an aneurysmal SVG.^214^ [64CU]- and [68Ga]-DOTA-Tyr3-octreotate ([64CU]- and [68Ga]-DOTATATE, respectively) bind to somatostatin type II receptors present on proinflammatory M1 macrophages, with high affinity.^215^ A prospective PET imaging study demonstrated that [68Ga]-DOTATATE can identify native coronary atherosclerotic plaques with greater specificity than 18-fluorine-fluorodeoxyglucose.^214^ The use of [68Ga]-DOTATATE to identify the in vivo contribution of inflammation to SVG disease is currently underway (ClinicalTrials.gov, NCT06800430).

## III Using multiomics technologies for improved therapeutic design

Multiomics approaches, which integrate genomics, transcriptomics, proteomics, metabolomics, and epigenomics, often at single cell and single-nuclei resolution, have the potential to provide a comprehensive understanding of cellular states and disease mechanisms. By capturing molecular interactions across multiple biological layers, these strategies enable the precise identification of pathogenic cell subsets and therefore, potential therapeutic targets. Such high-resolution profiling is particularly valuable for the development of cell-targeted advanced therapies, including RNA and gene therapies. Ultimately, multiomics integration enhances the ability to design interventions that are both mechanistically informed and tailored to specific cellular contexts, improving efficacy while minimizing off-target effects. Here, we describe how multiomics approaches are being used to identify disease-relevant cell subtypes in the context of vein graft failure.

Sun et al^216^ characterized the heterogeneity of harvested human saphenous vein tissue. The authors identified not only canonical endothelial cell and vascular smooth muscle cell gene signatures, but also previously undescribed disease-relevant subsets such as IL6hi proinflammatory endothelial cells, vascular smooth muscle cells, pericyte, and vein-specific fibroblast subsets such as ANGPTL7^+^ fibroblasts. These disease-associated cell states display transcriptional networks and ligand-receptor interactions, such as TRAIL and GDF15 in vascular endothelial cells and ANGPTL7^+^ fibroblasts, respectively, enriched in patients with diabetes and hypertension, suggesting a direct link between metabolic dysfunction and a targetable molecular milieu in the pregraft human saphenous vein wall.

These findings of native ungrafted human saphenous vein converge with spatial and single-cell data from studies such as Michaud et al,^217^ which leverage paired single-nuclei and spatial transcriptomics in a canine model of vein graft disease to map the immediate transcriptomic changes induced by vein harvest, distension and graft implantation. Adding a time dimension, this study profiles canine vein grafts immediately after distension and 24 hours after graft implantation to carotid circulation. The authors describe that graft injury does not result only from postimplantation arterial circulation exposure but is triggered early at vein harvest and distension. Key early transcriptional events include the upregulation of genes for cell proliferation, migration, and extracellular matrix remodeling in endothelial cells, vascular smooth muscle cells, and fibroblasts. Activated endothelial cells, endothelial cell subpopulations expressing mesenchymal markers (EndMT), proto-myofibroblasts, and myeloid cells quickly accumulate in damaged intimal and medial regions. Notably, the authors connect upregulation of canonical vascular remodeling, inflammatory genes and graft failure (eg, *IL-6*, *TGFBR1*, *SMAD4*, and *ADAMTS9*) to specific cell states and spatial regions (intima and media) of the grafted vein and reveal that these maladaptive gene regulatory networks are exacerbated by the hemodynamic implantation environment.

Similarly, a spatial transcriptomic study by McQueen et al^218^ provides a complementary view of the earliest transcriptional events triggered by arterial pressure exposure of vein grafts. Within just 4 hours of acute hemodynamic environment that mimics arterial circulation, the study identified 413 differentially expressed genes (372 upregulated, 41 downregulated) spanning classical pathways involved in early vein graft disease pathogenesis (eg, NF-κB, TNF, mitogen-activated protein kinase, and phosphoinositide 3-kinase/protein kinase B signaling) indicating a rapid onset of prophenotypic switch, proinflammatory and leukocyte-adhesive responses within the graft wall.

The endothelial cluster demonstrated strong induction of canonical inflammatory mediators (interleukin-6 (IL-6), interleukin-1 Beta (IL-1B), and C-X-C Motif Chemokine Ligand 8 (CXCL8)) and immediate-early genes such as Immediate Early Response 3 (*IER3*) and TNF Alpha Induced Protein 3 (*TNFAIP3*), suggesting endothelial activation and apoptotic stress responses under arterial shear stress-mimicking conditions. The smooth muscle cell cluster, comprising approximately 20% of mapped regions, upregulated IL-6 and LIF (Leukemia inhibitory factor), consistent with early phenotypic switching from a contractile to a synthetic state that underpins intimal hyperplasia in vein graft. In the adventitial fibroblast cluster, transcripts such as *CXCL8*, *CCL2* (C-C Motif Chemokine Ligand 2), *IL-6*, *IL-1B*, and intercellular adhesion molecule-1 were enriched, whereas *ACTA2* *(α smooth muscle cell actin, αSMA)* and other cytoskeletal genes were suppressed, akin to features of a proto-myofibroblast phenotype primed for extracellular matrix remodeling and migration. A spatially distinct hybrid endothelial cell-smooth muscle cell cluster expressing both endothelial and mesenchymal markers was identified within the inner wall, marking the onset of EndMT in this model of early vein graft remodeling.

In an integrative multiomics preprint by Kim et al,^219^ using a paired ex vivo culture model of 73 patient-derived saphenous vein samples, the team integrated bulk RNA sequencing, proteomics and spatial transcriptomics to capture the transcriptional and proteomic reprogramming that occurs within the first 7 days postharvest, modeling a critical window of injury response after graft implantation. Modeling vein graft disease, cultured saphenous vein segments developed a hyperplastic neointima characterized by *α*-SMA^+^ vascular smooth muscle cell accumulation, fragmentation of the internal elastic lamina and elastin degradation, whereas the luminal endothelium remained largely intact. Morphometric quantification confirmed a marked expansion of the neointimal area and a rise in the intima-to-media ratio, accompanied by fibroblast-to-myofibroblast conversion and smooth muscle cell phenotypic switching from a quiescent contractile to a synthetic, proliferative state—hallmarks of early intimal hyperplasia.

At the transcriptomic level, 2416 genes were upregulated and 1967 downregulated between day 0 and day 7 samples, reflecting a profound transcriptional pivot from contractility to inflammation and proliferation. Upregulated pathways included NF-κB and TNF signaling, cell-cycle regulation and stress response networks, featuring transcripts such as *IL-1B*, *IL-6*, *NFKB1/2*, *CDK1*, and *CCNB1* consistent with injury-induced inflammatory and proliferative remodeling. In contrast, genes central to vascular smooth muscle cell differentiation (*ACTA2*, *TAGLN*, *CNN1*, and *LMOD1*) and extracellular matrix integrity (*COL3A1*, *DCN*, *ELN*, and *FBLN1*) were significantly suppressed, alongside mitochondrial and fatty acid oxidation genes, suggesting induction of metabolic changes. Proteomic profiling reinforced these observations, identifying disrupted metabolic networks and upregulation of coagulation and hemostatic proteins, linking the early remodeling phase to a prothrombotic, proinflammatory microenvironment. Spatial transcriptomics adds a further layer of resolution, locating distinct fibroblast and vascular smooth muscle cell subpopulations that orchestrate neointimal hyperplasia. Trajectory mapping revealed transitions from contractile smooth muscle cells at baseline to synthetic and secretory fibroblast phenotypes expressing MMP2^+^/MMP14^+^, indicative of extracellular matrix degradation and remodeling. A novel LGALS3^+^/SPP1^+^ vascular smooth muscle cell subset emerged, marked by oxidative stress and extracellular matrix interaction signatures echoing phenotypes seen in human failed human grafts and diabetic vasculopathy. Collectively, these data outline a temporal and spatial hierarchy of cellular activation, where fibroblast-driven matrix degradation and vascular smooth muscle cell dedifferentiation converge to establish a maladaptive neointima niche. Patient stratification of the spatial transcriptomic datasets revealed biologically meaningful heterogeneity such as male saphenous veins exhibiting greater intimal thickening and higher intima/media ratios, whereas body mass index inversely correlates with neointimal hyperplasia in males but not females, suggesting sex- and metabolism-dependent differences in the vein wall responses to grafting.

Ni et al^220^ used an inducible lineage tracing strategy in a mouse model of vein grafting to map the origins, fates and functional contributions of Sca-1^+^ progenitor cells during vein graft remodeling. The work addressed a disputed question in a vein graft biology as to how discrete progenitor populations contribute to neointimal hyperplasia, adventitial remodeling and re-endothelialization after grafting, processes that collectively drive neointimal hyperplasia and graft failure over time. Single-cell RNA (ScRNA)-sequencing profiling of vein grafts at 4 weeks revealed a heterogeneous cellular landscape comprising endothelial cells, vascular smooth muscle cells, adventitial cells of mesenchymal origin and infiltrating leukocytes. Within this context, Sca-1^+^ cells emerged as key progenitors capable of generating both endothelial cells and vascular smooth muscle cells, but which was cell-origin and cell-location dependent. Recipient artery-derived Sca-1^+^ cells dominated the process of re-endothelialization, particularly in perianastomotic regions, accounting for approximately 75% of regenerated endothelial cells in these areas. These cells also contributed modestly to neointimal vascular smooth muscle cells (approximately 13%) and to the initiation of adventitial neoangiogenesis near the anastomotic regions. In contrast, donor vein-derived Sca-1^+^ progenitors predominantly committed to vascular smooth muscle cell differentiation within the neointima, driving intimal hyperplasia along the midportion of the graft and accounting for nearly 46% of *α*-SMA^+^ neointimal vascular smooth muscle cells. Although Sca-1 is specific to mice, the authors propose that CD34^+^ cells (often coexpressed with Sca-1 in mice) may serve as the functional human counterpart, potentially mediating similar multilineage contributions during human vein graft adaptation.

Ying et al^221^ using scRNA-sequencing, explored the cellular origins and molecular mechanisms that govern endothelial regeneration after vascular injury in mice. Using a combination of scRNA-sequencing, lineage tracing and functional mouse models of femoral artery injury and vein grafting, the authors identified a resident progenitor population marked by Sca-1^+^ population as a progenitor pool for endothelial cells during vascular repair. Lineage tracing profiled by scRNA-sequencing confirmed that Sca-1^+^ cells actively differentiated into endothelial cells after both femoral artery wire injury and inferior vena cava to carotid artery interposition vein graft model. Under ungrafted conditions, Sca-1^+^ cells were sparse within the endothelium, but their proportion increased significantly from approximately 12% to over 33% of the endothelial population in femoral arteries postinjury. A similar pattern was seen in the vein graft model, where Sca-1^+^ endothelial cells rose from 12% in uninjured inferior vena cava to nearly 40% at 4 weeks of graft remodeling. Importantly, validation experiments using targeted ablation of Sca-1^+^ cells markedly impaired endothelial regeneration, demonstrating that this progenitor pool is essential for re-endothelialization and vascular repair.

An integrative single-cell and metabolomic study by Gu et al^222^ provides insight into the cellular and metabolic contribution of perivascular adipose tissue (PVAT) to vascular remodeling. Using scRNA-sequencing of primary murine PVAT, the authors mapped a previously unappreciated diversity of PVAT-derived adipose stem cells and established their functional role as progenitors of smooth muscle differentiation and participation in neointima formation. Two transcriptionally distinct PVAT-derived adipose stem cell clusters were defined. Cluster 1 exhibited angiogenic and adipo-genic features expressing *Pecam1*, *Cd36*, *Fabp4*, and *Pparg* suggesting roles in vascular support and lipid homeostasis. In contrast, cluster 2 showed a myogenic transcriptional signature, expressing *Tgfbr2*, *Col3a1*, *Pdgfra/β*, and *Klf4* and pathway enrichment revealed upregulation of TGF-*β*, platelet-derived growth factor, and phosphoinositide 3-kinase/protein kinase B signaling, implicating it as a progenitor reservoir for vascular smooth muscle cell commitment and differentiation.

A multiomic study by Decano et al^223^ identified peroxisome proliferator-activated receptor-*α* (PPAR*α*) as a central regulator of macrophage metabolism and vascular inflammation in vein graft remodeling. Through an integrative workflow combining global proteomics, high-dimensional network clustering in 4 weeks Ldlr^−/−^ murine vein grafts and scRNA-sequencing in primary macrophages, the study delineated a molecular atlas of lesion-prone and lesion-resistant regions, revealing that metabolic dysregulation and inflammatory activation converge at the neointimal-adventitial interface—a critical location for graft failure. Vascular layer-specific proteomics uncovered profound differences between neointimal and adventitial regions. Lesion-positive areas were enriched for matrix remodeling and inflammatory pathways, including upregulation of MMPs, TNF/NF-κB, and IL-1*β* signaling, whereas lesion-free tissues retained high expression of mitochondrial metabolic enzymes and fatty acid oxidation genes, many in the PPAR*α* transcriptional network. Mechanistically, scRNA-sequencing analysis of macrophages revealed that PPAR*α* orchestrated a metabolic rewiring of macrophages, shifting them from glycolytic, proinflammatory states toward oxidative, reparative phenotypes. ScRNA-sequencing further resolved macrophage heterogeneity, identifying a distinct proinflammatory subcluster whose transcriptional signature defined by IL-1*β*, SPP1 (Secreted Phosphoprotein 1), and CCL2 were selectively repressed with pharmacological treatment using pemafibrate, validating PPAR*α*’s role in macrophage state modulation at single-cell resolution.

**Fig. 3:**
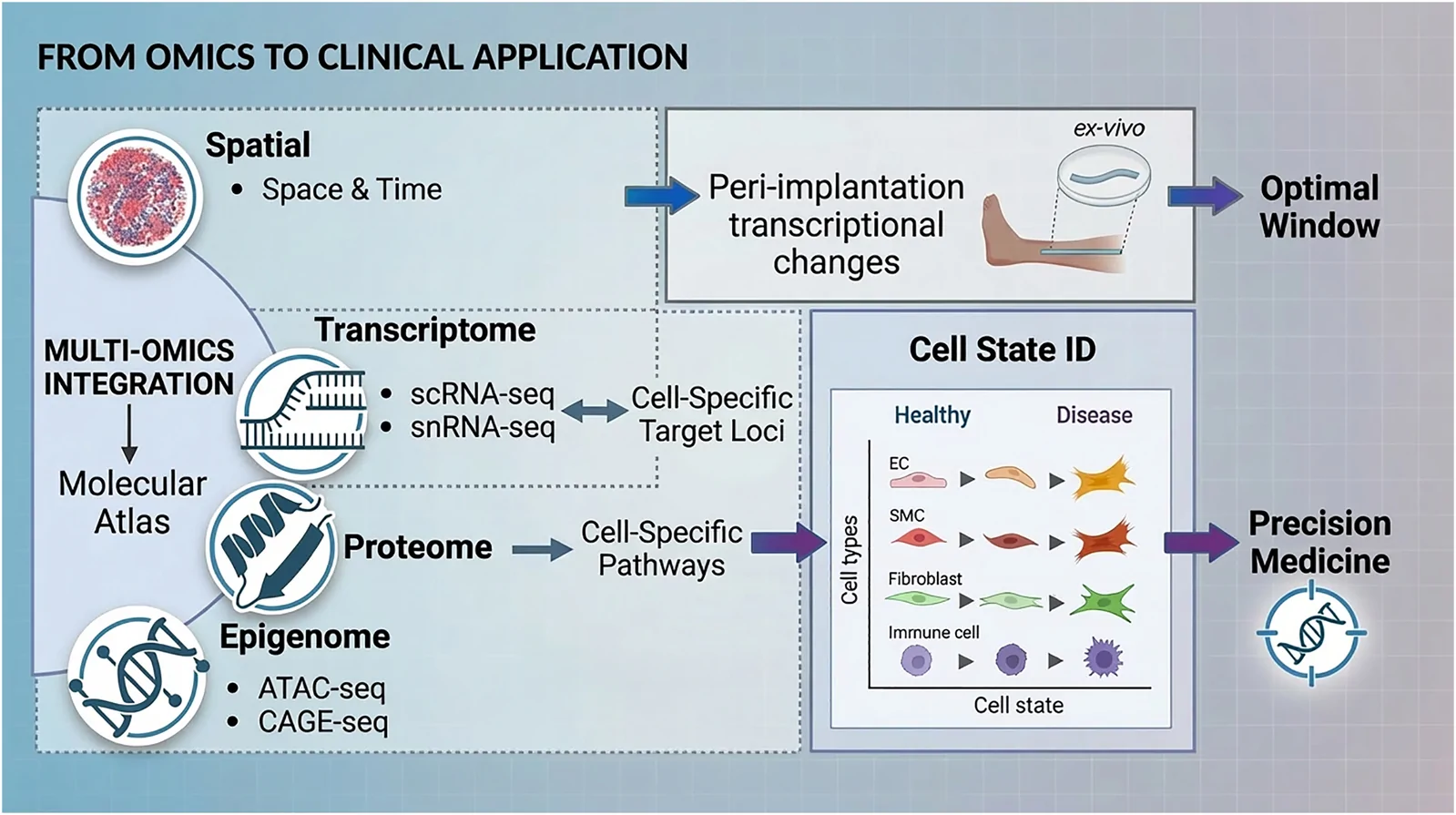
Omics technologies and the design of advanced gene and RNA therapies. Multiomics, or “omics,” is a collective term for methodologies that enable an unbiased and comprehensive analysis of biological systems. These techniques include, but are not limited to, transcriptomics, proteomics and epigenomics. Multimodal data integration provides a powerful and in-depth understanding of not only basic biology but also the pathobiology of disease. It can also serve as a discovery platform for specific genes, gene regulatory networks, or disease-associated cell states and pathways, any of which can be selectively targeted to support a precision-driven therapeutic strategy. Recent omics studies example (spatial transcriptomics, single nucleus RNA sequencing [snRNA-seq]) in vein grafting have revealed that the optimal window for transcriptional disease changes occurs during the peri-implantation stage. Such disease insight and window of therapeutic opportunity together with omics-derived specific gene or cell targets can drive the next generation of advanced vein graft therapies. ATAC-seq, assay for transposase-accessible chromatin with sequencing; CAGE-seq, cap analysis gene expression sequencing; EC, endothelial cell; SMC, smooth muscle cell.

Multiomics findings to date consistently show that the greatest divergence in cell state and signaling occurs immediately after graft harvest and in the early timepoint of graft exposure to arterial flow. As a result, the optimal intervention window is the peri-implantation period, when transcriptional malleability is highest and maladaptive cascades can still be redirected toward graft adaptation rather than progression to occlusion and failure. This “window of opportunity” emphasizes the importance of the rapid application of targeted advanced therapeutics. Translating mechanistic knowledge into clinical interventions may use 2 parallel strategies. First, a discovery phase, omics-driven mapping highlights target loci (Fig. 3) using Cap Analysis of Gene Expression-sequencing datasets from vein graft models.

**Fig. 4:**
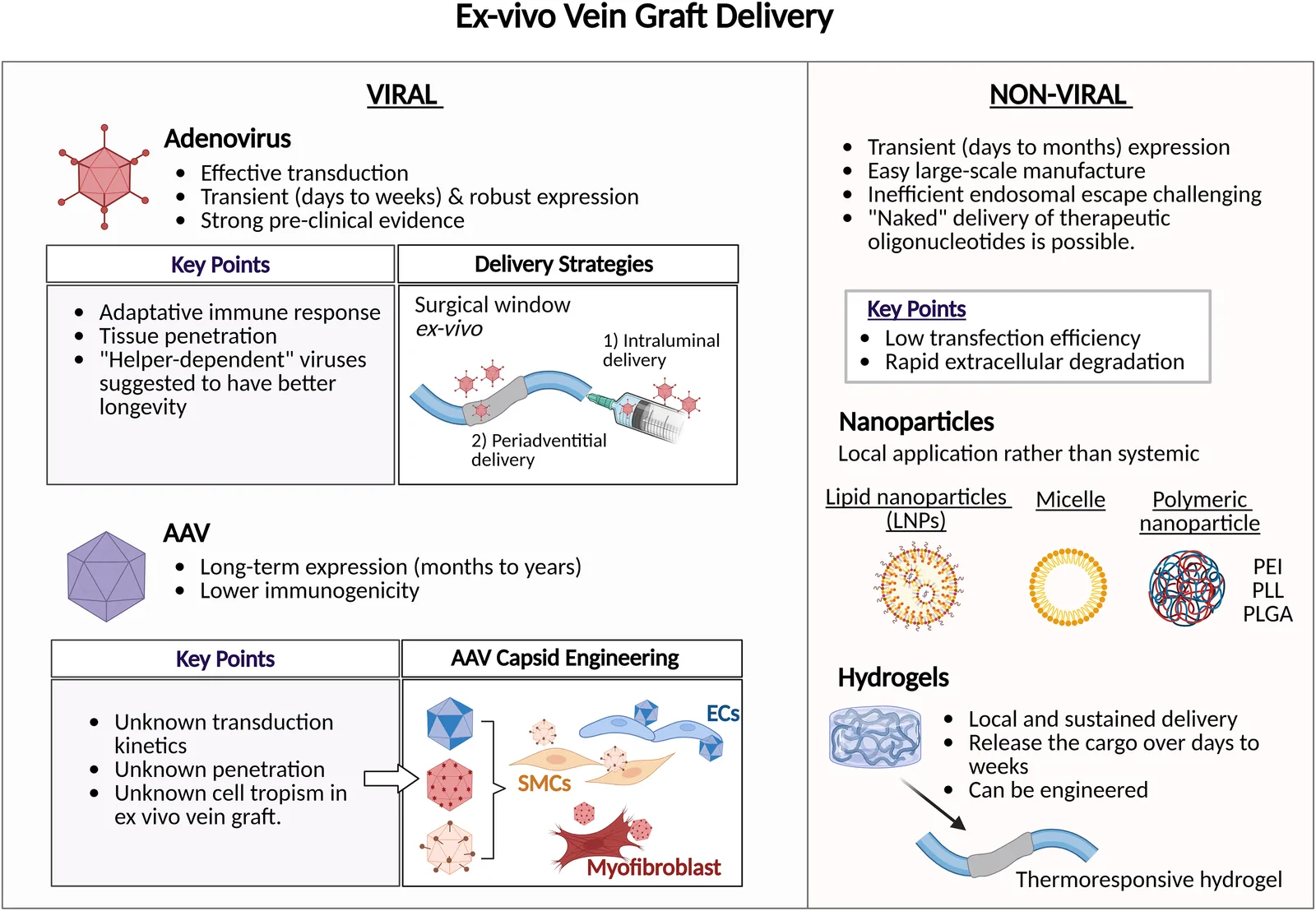
Delivery modalities for the prevention of vein Graft failure. Delivery of advanced therapies remains a major challenge that limits the clinical translation of many promising preclinical studies. Both viral and nonviral delivery systems have strengths and limitations that must be considered when selecting the best strategy. Adenoviruses offer high but transient (days to weeks) transduction efficiency and have been strongly validated in preclinical studies. However, they can generate an adaptive immune response, and their clinical success depends on cellular tropism and tissue penetration. AAVs exhibit lower immunogenicity compared with adenoviruses and offer long-term expression (months to years), but their transduction efficiency is limited by AAV penetration and cellular tropism. Engineering of AAV capsids is a promising approach to overcome these limitations. Nonviral delivery systems, such as LNPs, micelles, polymeric nanoparticles (polyethylenimine [PEI], poly-L-lysine [PLL], and poly(lactic-co-glycolic acid) [PLGA]), and hydrogels, are emerging as important delivery vectors, particularly for oligonucleotide-based moieties such as siRNA and ASOs because of their low immunogenicity, tunability, scalability, and compatibility with intraoperative, perivascular application. However, inefficient endosomal escape is a key limitation, although evidence is emerging suggesting “naked” delivery of nucleic acid-based therapies to the vasculature is possible. EC, endothelial cell; SMC, smooth muscle cell.

Second, a delivery phase, where modulation of target loci can be achieved through the development of targeting vectors (eg, adeno-associated viral [AAV] vectors and nanoparticles) that are locally delivered to the graft at harvest or peri-implant (Fig. 4). Local application, whether ex vivo (soaking or perfusing the graft) or via injectable biomaterials ensures spatially restricted exposure. Further largescale profiling of vein graft transcriptome and epigenome in clinically relevant animal models are needed, together with innovation in synthetic regulatory elements are necessary to minimize off-target effects and maximize clinical precision, ultimately bringing CABG surgery into the realm of real precision medicine.

## IV Designing next-generation therapeutics and materials for the failure of vein graft disease

### A RNA therapeutics

Advanced gene and RNA therapies typically function through the modulation of mRNA or noncoding RNA levels, depending on whether the therapeutic benefit stems from the increase or decrease in target levels. Methods to reduce the levels of target RNAs include short interfering RNAs (siRNAs), short hairpin RNAs, ASOs, and other small ASO-like compounds such as morpholino antisense oligomers and peptide nucleic acid molecules. siRNAs and short hairpin RNAs utilise the endogenous RNA interference pathway, in which short RNA species can associate with the RNA-induced silencing complex to mediate the degradation of target RNAs, which are identified based on base complementarity between the siRNA and the target RNA. siRNAs are now a relevant therapeutic modality toward cardiovascular disease, including for the prevention of vein graft failure. Significant preclinical research is emerging that demonstrates that targeting cell-specific, noncoding genes with key roles in the development of vein graft failure using siRNA is a robust and clinically-relevant approach to treat vein graft disease (Table 3).^169^^,^^179^^,^^181^^,^^182^^,^^224^, ^225^, ^226^, ^227^, ^228^, ^229^, ^230^, ^231^, ^232^, ^233^, ^234^, ^235^, ^236^ ASOs are similarly emerging as a promising therapeutic modality toward cardiovascular disease,^237^ although this has yet to extend to CABG or PABG failure.

**Table 3:** Translational targets for vein graft failure

Target Gene/Pathway	Therapeutic Modality	Model/Setting	Study Type	Key References
TIMP-3 (tissue inhibitor of metalloproteinase-3)	Adenovirus	Porcine autologous vein grafts treated ex vivo	Preclinical	George et al^181^^,^^182^
PTEN (phosphatidylinositol 3-kinase)	Adenovirus	Canine vein graft model, rat carotid model	Preclinical	Hata et al^225^; Huang et al^226^
STAT-3 (signal transducer and activator of transcription 3)	siRNA	Rat jugular vein: carotid artery bypass graft	Preclinical	Sun et al^227^
iNOS (inducible nitric oxide synthase)	Plasmid	Rabbit vein graft model	Preclinical	Meng et al^228^
p53 overexpression	Adenovirus	Human saphenous vein organ culture	Preclinical	Wan et al^229^
Constitutively active retinoblastoma (ΔRb) mutant	Adenovirus	Rabbit vein graft model	Preclinical	Schwartz et al^230^
C-type natriuretic peptide	Adenovirus	Rabbit vein graft model	Preclinical	Ohno et al^231^
SMILR	siRNA (BHF7)	Human saphenous vein organ culture	Preclinical	Brown et al^224^
miRNA-21	Antisense oligonucleotide	Rat carotid injury, porcine saphenous vein-carotid interpositional grafting, human saphenous vein organ culture	Preclinical	Ji et al^169^; McDonald et al^232^
miR-323a-3p	miRNA mimic	Human saphenous vein smooth muscle cells, human saphenous vein organ culture	Preclinical	Rodor et al^179^
miR-449b-5p	miRNA mimic	Human saphenous vein smooth muscle cells, human saphenous vein organ culture	Preclinical	Rodor et al^179^
miR-491-3p	miRNA mimic	Human saphenous vein smooth muscle cells	Preclinical	Rodor et al^179^
miR-892b	miRNA mimic	Human saphenous vein smooth muscle cells	Preclinical	Rodor et al^179^
miR-1827	miRNA mimic	Human saphenous vein smooth muscle cells	Preclinical	Rodor et al^179^
miR-4774-3p	miRNA mimic	Human saphenous vein smooth muscle cells	Preclinical	Rodor et al^179^
miR-5681b	miRNA mimic	Human saphenous vein smooth muscle cells	Preclinical	Rodor et al^179^
SMAD3 (SMAD family member 3)	Lentivirus shRNA	Porcine arteriovenous fistula model	Preclinical	Xu et al^233^
TIMP-3 (tissue inhibitor of metalloproteinase-3)	Adenoviral vector (Ad5.CMVTO.TIMP-3)	Human saphenous vein ex vivo (PROTECT trial)	Clinical	ISRCTN^234^
E2F transcriptional factor family	Oligonucleotide decoy (edifoligide)	Human saphenous vein grafts ex vivo (PREVENT trials)	Clinical	Alexander et al^235^; Conte et al^236^

shRNA, short hairpin RNA.

Key preclinical and clinical studies using gene and RNA therapies to modulate genes implicated in vein graft failure.

Methods to increase the levels of genes that are noted to be downregulated in disease include synthetic oligonucleotide mimics. These have shown the greatest promise with microRNA mimics, which are powerful tools to increase the levels of disease-relevant miRNAs intracellularly.^238^ MicroRNAs are a pervasive noncoding RNA species that can target several mRNAs in a large network, meaning it is possible to effect a broad range of targets through the targeting of a single miRNA. For example, Rodor et al^179^ identified several novel miRNAs that have antiproliferative functions across vascular smooth muscle cells from multiple vascular beds. When these microRNAs are increased by the exogenous delivery of miRNA mimics, smooth muscle cell proliferation is significantly reduced both in vitro and ex vivo.^179^ These studies, and others, demonstrate the potential of miRNA mimics as powerful therapeutics for the prevention of vein graft failure, as well as many other pathologies (Table 3).

### B Adenoviral vectors: Promise, mechanisms, and limitations

Adenoviral (Ad) vectors have been the cornerstone of vascular gene transfer for more than 2 decades, owing to their high transduction efficiency across both dividing and nondividing cells. They remain particularly attractive for ex vivo vein graft modification, where transient but robust transgene expression can be harnessed without systemic exposure. However, despite compelling preclinical results (Table 3), translation to durable clinical efficacy in humans has been inconsistent, reflecting the inherent immunogenicity and transient expression profile of first-generation Ad systems.

#### 1 Preclinical evidence of efficacy

Adenoviral gene transfer has demonstrated robust therapeutic potential across diverse models of vein grafting and arterial injury, where early pathological events, including vascular smooth muscle cell proliferation, inflammation, extracellular matrix degradation, and lipid deposition, drive neointimal hyperplasia and eventual graft failure. In these settings, adenoviral vectors have been used to deliver a range of mechanistically targeted genes, each addressing a distinct component of vascular remodeling. Among the most compelling examples is inhibition of microRNA-21 (miR-21), a key regulator of vascular smooth muscle cell proliferation and survival that is consistently upregulated after vein graft implantation. In rat vein graft models, adenoviral delivery of miR-21 sponges significantly reduced neointimal hyperplasia by restoring phosphatidylinositol 3-kinase and Spry1 levels and thereby suppressing protein kinase B-mediated synthetic switching of vascular smooth muscle cells.^239^ Complementing this antiproliferative strategy, metabolic reprogramming has been achieved using helper-dependent adenoviruses encoding apolipoprotein A-I, which produced sustained transgene expression in hyperlipidemic rabbits and led to marked reductions in intimal lipid accumulation, macrophage infiltration, and foam-cell formation—effects attributed to enhanced cholesterol efflux and the reduced inflammatory footprint of gutless adenoviral vectors.^240^

Additional inflammatory targets have broadened the mechanistic scope of adenoviral therapy: expression of soluble vascular cell adhesion protein-1 reduced leukocyte adhesion and endothelial activation; blockade of MCP-1 (Monocyte Chemoattractant Protein-1) signaling using the dominant-negative mutant 7ND attenuated macrophage recruitment and intimal thickening in canine grafts; and adenoviral fibromodulin restored extracellular matrix organization and suppressed neointimal hyperplasia in human saphenous vein organ culture.^241^^,^^242^ Together, these preclinical programs underscore a consistent principle: although adenoviral expression is inherently transient, short-duration, high-intensity gene delivery during the early postoperative window is sufficient to substantially reprogram the trajectory of vein graft remodeling, providing a strong mechanistic rationale for continued refinement of adenoviral platforms in translational vein graft therapy.

Matrix-directed approaches have been particularly influential, with TIMP-3 emerging as one of the most clinically advanced candidates. Adenoviral TIMP-3 overexpression inhibited MMP-driven extracellular matrix turnover, curtailed vascular smooth muscle cell migration, and robustly suppressed neointimal hyperplasia ex vivo and in porcine vein grafts in vivo.^181^^,^^182^

#### 2 Clinical evidence

The evolution of vein graft failure prevention has transitioned from fundamental molecular biology to a landmark clinical milestone through the investigation of TIMP-3. Early preclinical evidence established that the adenoviral-mediated overexpression of TIMP-3 effectively inhibits MMP activity and induces apoptosis in vascular smooth muscle cells.^180^ When translated to a porcine model (specifically the pig saphenous vein-to-carotid artery interposition graft), adenoviral gene transfer demonstrated a reduction in neointima formation.^181^ Long-term assessments at the 3-month mark confirmed that recombinant adenovirus TIMP-3 provides sustained retardation of intimal thickening, laying the groundwork for ex vivo gene therapy in a clinical setting.^182^

Building upon this preclinical evidence base, the Placebo-contROlled Trial of Ad5.CMVTO.TIMP-3 to prEvent Coronary artery bypass grafT failure (PROTECT) study represents a pioneering effort in translational cardiovascular medicine. This first-in-human, prospective, randomized, open-label, dose-ranging phase 1 clinical trial evaluates a novel gene therapy strategy designed to enhance the long-term patency and durability of SVGs (ISRCTN43650325). The therapeutic strategy uses a viral vector to deliver the TIMP-3 gene directly into the SVG lumen, targeting the underlying mechanisms of pathological tissue remodeling that cause graft stenosis and occlusion.

**Fig. 5:**
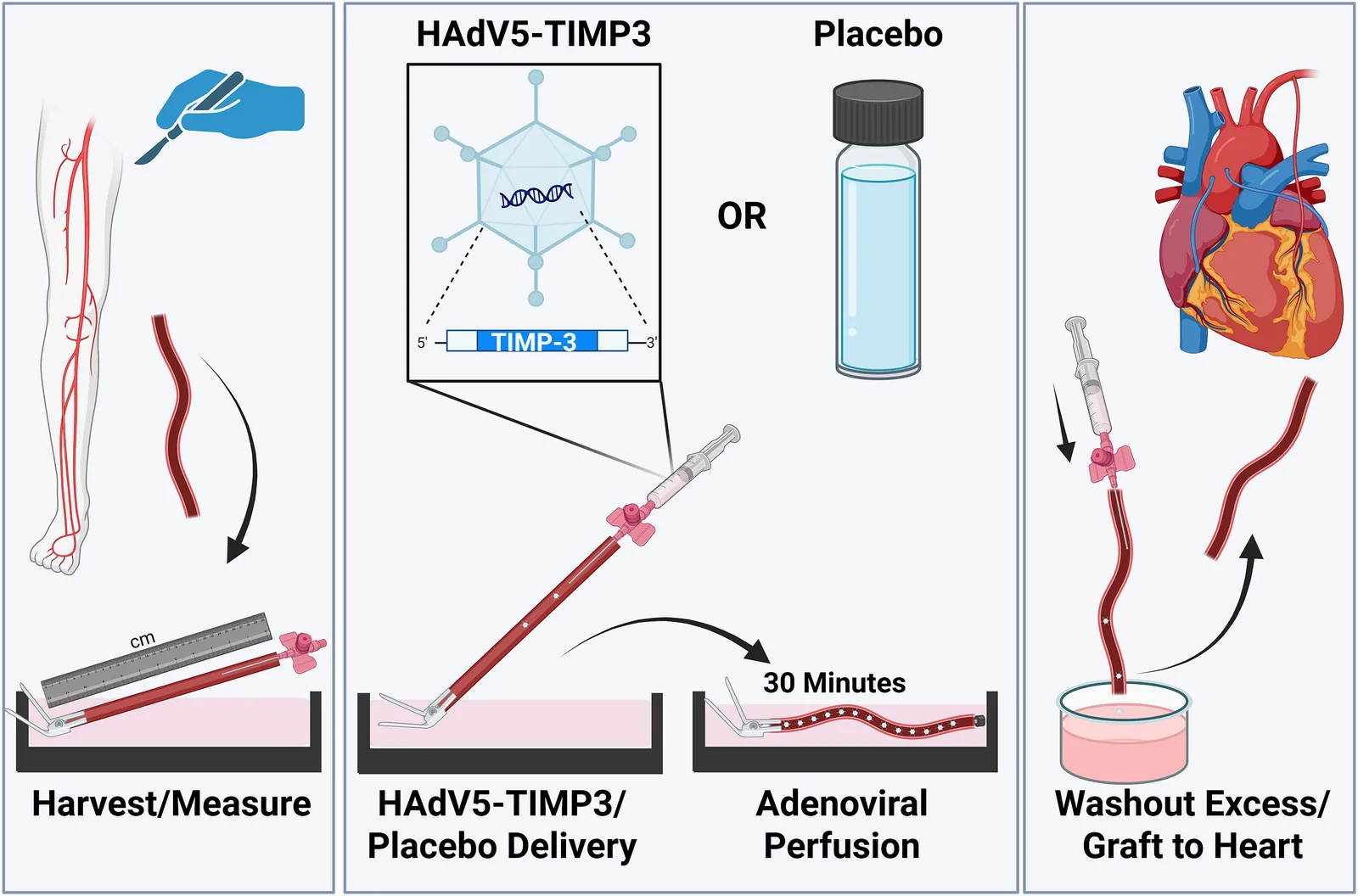
PROTECT clinical trial delivery. Outline of the study design for the phase 1 Placebo-contROlled Trial of Ad5.CMVTO.TIMP-3 to PROTECT. In brief, the saphenous vein graft is harvested and measured, and the corresponding dose of Ad5.CMVTO.TIMP-3 is calculated. Ad5.CMVTO.TIMP-3 is instilled through the lumen of the vein graft via a vein catheter to displace any existing air and fluids. The distal and proximal ends of the vein are then sealed to retain the solution within the lumen of the vein for a 30-minute incubation period. After this, the graft is gently flushed with 20 mL sterile heparinized 0.9% sodium chloride (to remove any virus that has not transduced the graft). The prepared grafts will then be transferred to the surgeon for aorto-coronary anastomosis using standard techniques.

The delivery protocol of the PROTECT trial is designed to maximize therapeutic efficacy while ensuring patient safety through highly localized application. The saphenous vein is harvested and immediately treated ex vivo with the viral vector before surgical implantation. In this trial, the randomization procedure involves allocation of the Ad5.CMVTO.TIMP-3 gene therapy to 1 of 2 vein grafts, with the other vein graft allocated to receive placebo (Fig. 5). In this way, the vein grafts rather than the participant receive treatment at random, and all participants will receive active treatment. Moreover, this mitigates the confounding effects of the method of saphenous vein harvesting or systemic factors on subsequent graft failure given the within person comparison. This approach allows for direct and efficient delivery to the target vascular tissue, ensuring that the overexpression of TIMP-3 occurs precisely where it is required to prevent neointimal hyperplasia. Crucially, this ex vivo methodology minimizes systemic exposure to the viral vector, reducing the risk of off-target effects and systemic inflammatory responses.^234^

Safety evaluations are typically the primary objective of a phase 1, first-in-human trial. Therefore, the primary outcome of this phase 1 trial is dose-limiting toxicity reflected by the incidence of treatment emergent adverse events. SVG patency is both a safety and efficacy outcome and the participants will undergo 3 cardiac gated multidetector coronary computed tomography angiography scans postrandomization. The imaging time-points are days 5 to 7, 6 weeks, and 12 months. The coronary computed tomography angiography endpoints include SVG occlusion at 12-months; other endpoints include SVG with diameter stenosis >50%, SVG with diameter stenosis >50% or occlusion, and SVG revascularization. Custom-developed imaging software will be used to quantify vein graft lumen diameter and categorize the stenosis severity (% of the reference lumen diameter) of any lumen narrowing. SVG area, lumen area, wall thickness, length, and related volumes will also be quantified.

The first cohort of patients have undergone CABG surgery using the TIMP-3-treated vein or placebo and progress in the trial is being overseen by a Safety Review Committee in conjunction with the Sponsor.

The PROTECT trial, supported by the Medical Research Council and the British Heart Foundation, signifies a potential shift in the management of CABG surgery. If safety is confirmed in the phase 1 trial, then the next step will be to assess efficacy and safety in a larger, phase 2 clinical trial. If this future trial provides evidence of efficacy and safety, then a phase 3 clinical outcomes trial would be finally required. By addressing the biological drivers of graft failure—rather than relying solely on systemic pharmacological interventions—TIMP-3 gene therapy aims to reduce the incidence of late-stage graft failure, improve long-term life expectancy, and decrease the necessity for high-risk repeat revascularization procedures. As the study progresses, it remains a focal point for the integration of molecular therapeutics into cardiac surgical practice, marking a definitive step toward personalized, biology-driven cardiovascular surgery.^243^

A previous clinical effort using the antisense compound AVI-5126 was terminated early because of lack of demonstrable benefit (ClinicalTrials.gov, NCT00451256). These outcomes underscored not only the limitations of individual molecular targets but also intrinsic constraints of adenoviral delivery in the human venous conduit. A major translational barrier is innate immune activation, which is well documented in preclinical models. Newman et al^244^ showed that even “empty” HAdV5 vectors induce endothelial activation, macrophage recruitment, and neointimal formation in rabbit arteries via Toll-like receptor- and NF-κB–dependent pathways.^244^ Such inflammatory responses can directly oppose the intended therapeutic effects. Compounding this, pre-existing neutralizing antibodies against common HAdV5 serotypes reduce transduction efficiency and shorten the already transient expression window, particularly in older and previously exposed populations.

Much of the clinical variability reflects differences in cellular tropism and tissue penetration, which have been extensively characterized across in vitro, ex vivo, and in vivo systems. In vitro, human vascular smooth muscle cells and endothelial cells are readily transduced by Ad5, though efficiency is strongly influenced by CAR expression, which declines after vascular injury and varies among venous versus arterial beds.^245^ Alternative serotypes such as HAdV35 and HAdV49, which use CD46 rather than the chimeric antigen receptor (CAR) for entry, have demonstrated substantially higher transduction of human vascular smooth muscle cells and endothelial cells and may partially circumvent pre-existing immunity.^246^ Ex vivo human saphenous vein studies consistently show preferential medial transduction, with adenovirus penetrating the graft wall from the luminal surface under controlled dwell or pressure-assisted conditions. This pattern aligns with the distribution of the target cell population, vascular smooth muscle cells driving neo-intimal hyperplasia, but creates limited access to the adventitia unless vector is applied peri-vascularly.^182^^,^^247^^,^^248^

In vivo, the model and route of delivery further shape cellular targeting. Intraluminal exposure favors endothelial and medial transduction but is constrained by rapid washout and endothelial barrier recovery. In contrast, periadventitial or “outside-in” delivery, achieved via topical gels, wraps, or injections, achieves deeper penetration into the adventitia and media and avoids luminal exposure, making it highly compatible with ex vivo graft preparation during CABG. These delivery-specific differences partly explain why results in animal models (where luminal injury is often induced) do not always translate directly to human grafts, which are typically implanted with relatively intact endothelium.^244^^,^^247^

Collectively, the clinical experience to date indicates that the biological potential of adenovirus is not in question with numerous preclinical studies showing consistent and robust activity, but success in humans depends on precise alignment of vector tropism, immune profile, and delivery strategy with the unique structural and immunological milieu of the vein graft. This has motivated the development of next-generation adenoviral platforms (rare-serotype capsids, helper-dependent systems) and refined ex vivo/periadventitial application techniques to more effectively exploit the narrow therapeutic window during early graft adaptation.

### C Adeno-associated viral vectors: Durable expression and emerging mural cell tropism

Compared with adenovirus, recombinant AAV offers a more favorable balance between durability of expression and immunogenicity and has therefore become the dominant platform for in vivo cardiovascular gene therapy. AAV is nonenveloped, largely nonintegrating, and elicits relatively modest innate and adaptive immune activation at clinically relevant doses, enabling transgene expression for months to years in postmitotic tissues.^249^, ^250^, ^251^ Recent consensus statements and reviews from highlight AAV as the leading vector for cardiac and vascular indications, while also emphasizing unresolved safety and dose-limiting immunogenicity issues.^252^^,^^253^ For vein graft disease, where intimal hyperplasia and accelerated atherosclerosis unfold over years rather than weeks, this kinetic profile is, in theory, attractive: a single intraoperative or early postoperative intervention could, in principle, provide long-term modulation of endothelial, smooth muscle, or mural cell behavior throughout the lifespan of the graft.

Early work comparing AAV with Ad in large vessels already hinted at these advantages. In rabbit carotid and iliac arteries, Gruchala et al^254^ showed that AAV mediated stable reporter expression in medial smooth muscle cells and adventitial cells for at least 12 to 16 weeks, whereas adenoviral expression peaked early and declined rapidly, and was accompanied by more pronounced leukocyte infiltration and luminal narrowing. These data suggested that AAV is intrinsically better suited to chronic vascular remodeling than to acute, “hit-and-run” applications. However, native serotypes exhibit relatively poor transduction of quiescent vascular smooth muscle cells and variable penetration across the vein graft wall. This has motivated an extensive program of capsid engineering and peptide display to retune AAV tropism toward discrete vascular cell populations that are mechanistically central to vein graft failure. One of the earliest demonstrations of smooth-muscle–selective targeting was provided by Work et al,^255^ who inserted the EYHHYNK peptide into the AAV2 capsid. In primary human venous and arterial smooth muscle cells, EYH-modified AAV2 increased transduction efficiency up to ∼70-fold compared with wild-type AAV2 at short, clinically relevant exposure times, while leaving endothelial cells largely untransduced.^255^ This in vitro and ex vivo selectivity directly maps onto the medial and neointimal vascular smooth muscle cell populations that drive intimal hyperplasia in vein grafts.

More recently, high-throughput in vivo library screening has refined this approach and produced capsids with even more pronounced mural specificity. Ramirez et al^256^ used an AAV9-based random heptapeptide library and iterative in vivo selection in mice to identify an AAV9 capsid displaying the peptide sequence PRPPSTH (AAV-PR), a capsid variant that exhibits striking preference for pericytes and vascular smooth muscle cells in the brain vasculature. After systemic administration, AAV-PR achieved dense labeling of *α*SMA^+^ pericytes on small-caliber microvessels and SM22*α*^+^ vascular smooth muscle cells in large arteries, including the aorta and coronary arteries, with relatively modest endothelial transduction. The same capsid efficiently transduced primary human brain pericytes in vitro. Although developed for neurovascular indications, the mechanistic implications for vein grafts are clear: the pericyte-like and synthetic vascular smooth muscle cell subpopulations that emerge within the adventitia and neointima after graft implantation could, in principle, be targeted selectively by AAV-PR–like capsids to deliver antiproliferative, proresolving, noncoding or protein-coding RNA-based sequences without compromising re-endothelialization.

Complementary capsid engineering now addresses the endothelial compartment. Krolak et al^257^ used a barcoded in vivo selection strategy to generate AAV-BI30, an AAV9-derived capsid that transduces >80% of arterial, capillary, and venous endothelial cells throughout the central nervous system at relatively low systemic doses (∼10^11^ vg per mouse), while evading most parenchymal cell types.^257^ AAV-BI30 also transduced human brain microvascular endothelial cells in vitro, demonstrating cross-species conservation of tropism. Although this work is central nervous system-focused, it provides a blueprint for engineering endothelial-tropic capsids that might be retasked to target activated vein graft endothelium, for example, to deliver endothelial nitric oxide synthase, thrombomodulin, or junction-stabilizing proteins to restore barrier function and reduce early thrombosis and leukocyte recruitment. In parallel, Schröder et al^258^ recently applied AAV9 capsid library screening with barcoded readouts directly on primary murine aortic vascular smooth muscle cells and human aortic tissue ex vivo, identifying an RFTEKPA-bearing capsid with ∼70-fold higher transgene expression in vascular smooth muscle cells than wild-type AAV9 and robust transduction across the full thickness of the human aortic wall. This vector effectively targets the media and inner adventitia, precisely the cellular zones that dominate midphase and late-phase vein graft remodeling, whereas again only modestly engaging the endothelium.

From a vein graft perspective, these mural-tropic and endothelial-tropic AAVs can be viewed as complementary tools to interrogate and therapeutically manipulate the distinct cell populations that underlie the temporal phases of graft failure. AAV variants targeted to vascular smooth muscle cells or pericytes could deliver cell-cycle inhibitors, matrix-modifying enzymes, or miRNA/lncRNA modulators to restrain intimal hyperplasia and adverse matrix remodeling in the intermediate and late phases, whereas acute-acting strategies (Ad, nonviral RNA delivery) might be better aligned with very early injury and thrombo-inflammatory events. However, no AAV-based therapy has yet been tested in a dedicated human vein graft trial and most evidence remains confined to arterial injury models, ex vivo human vein segments, or other vascular beds.

### D Nonviral delivery systems in vein graft disease: Platforms, models, and cell-specificity

Nonviral vectors have emerged as attractive alternatives to viral platforms for local gene and RNA delivery in the vein graft setting. Their principal advantages, lower immunogenicity, relative ease of large-scale manufacture, and avoidance of genomic integration are balanced by the need to overcome inherently lower transfection efficiency and more rapid extracellular degradation. In the context of vein graft disease, nonviral systems have been evaluated primarily as perivascular or ex vivo delivery vehicles for siRNAs, ASOs, plasmid DNA and microRNA mimics, to suppress neointimal hyperplasia, inflammation, and early thrombosis. Southerland et al,^243^ in their comprehensive review of gene therapy for vein graft disease, highlighted nonviral approaches as particularly well suited to the surgical window when the graft is accessible outside the body and the target wall layers can be directly coated or soaked while minimizing systemic exposure. Despite strong mechanistic rationale and robust preclinical responses, the clinical efficacy of nanoparticle-mediated gene therapy for vein graft failure has so far been limited.

#### 1 Clinical evidence

The most definitive test, the PREVENT IV trial, evaluated ex vivo delivery of the E2F transcription factor decoy edifoligide to human SVGs during CABG. Although designed to inhibit vascular smooth muscle cell proliferation, no reduction in graft failure was observed at 12 to 18 months (45.2% vs 46.3%; odds ratio = 0.96; *P* = .66).^235^ Despite extensive preclinical evidence that periadventitial drug delivery can effectively suppress intimal hyperplasia, successful translation into clinical practice has yet to occur. Only a limited number of human studies, largely focused on arteriovenous access rather than arterial bypass grafts, have tested these platforms. A trial of a rapamycin-eluting mesh (ClinicalTrials.gov, NCT01033357) ended early after investigators observed higher infection rates in the treatment arm. Another program evaluating a VEGF-D Ad construct delivered within collagen collars advanced to Phase III (ClinicalTrials.gov, NCT00895479) but was discontinued for nonefficacy-related strategic reasons. Attempts to enhance patency using perivascular endothelial cell implants, as in the V-HEALTH phase 1/2 trial, did not yield improvements in primary or secondary outcome.^259^ More recently, recombinant human pancreatic elastase applied around the venous anastomosis showed no overall benefit in early-phase testing (ClinicalTrials.gov, NCT01001351), although a subset of patients receiving radiocephalic fistulas experienced better 3-year patency, prompting further phase 3 evaluation (ClinicalTrials.gov, NCT02110901 and NCT02414841).^260^ Overall, no periadventitial delivery method has yet demonstrated clinical efficacy, highlighting a substantial translational gap between promising experimental data and durable human benefit.

#### 2 Nanoparticles

Among chemical systems, cationic lipids and lipid nanoparticles (LNPs) have become the workhorses for nucleic-acid delivery in cardiovascular models. Although most clinical experience with LNPs comes from liver-targeted siRNA drugs such as patisiran and inclisiran, the same design principles, ionisable lipids, polyethylene glycol-lipids for circulation time, and helper lipids for endosomal escape have been adapted for vascular applications, where LNPs are applied locally rather than systemically. Recent reviews of organic nanoparticles in cardiovascular disease summarize a growing body of work using lipid-based, polymeric and micellar carriers to deliver siRNAs, miRNA mimics and plasmid DNA to endothelial cells and vascular smooth muscle cells, with demonstrable effects on proliferation, inflammation, and thrombosis in arterial injury and graft models.^261^^,^^262^ Although vein graft-specific LNP studies are still rare, the rapid clinical maturation of lipid-based RNA delivery is likely to influence future graft-targeted strategies.

Cationic polymers and polymeric nanoparticles represent a second major nonviral class. Classic materials such as polyethylenimine and poly-L-lysine efficiently condense DNA and siRNA into polyplexes, but dose-dependent toxicity has driven a shift toward biodegradable polymers, notably poly(lactic-co-glycolic acid). In an important proof-of-concept study, Nishio et al^263^ developed miR-145–loaded poly(lactic-co-glycolic acid) nanoparticles and applied them ex vivo to rat carotid arteries; miR-145 delivery shifted vascular smooth muscle cells from a proliferative to a contractile phenotype, increased expression of MYH11 and ACTA2, and significantly reduced neointimal thickness after injury. Polymeric carriers have also been used to deliver plasmid DNA in vein graft models; for example, nanoparticle-mediated delivery of arresten cDNA in a rat jugular vein–carotid artery graft model reduced neointimal area and improved lumen preservation, underscoring the potential for nonviral plasmid delivery to alter vein graft remodeling.^264^

#### 3 Hydrogels

For local, sustained delivery around the graft, hydrogel, and periadventitial depot systems have been particularly influential. Thermoresponsive and photo-crosslinked hydrogels can be applied as liquids to the graft surface and then cured in situ, forming a depot that releases cargo over days to weeks—the critical period for early vein graft adaptation. In a rat carotid balloon-injury model, PEG-based hydrogels covalently modified with S-nitrosocysteine released NO for ∼24 hours and reduced neointima formation by ≈75% at 14 days, while also promoting endothelial recovery and reducing platelet adhesion.^265^^,^^266^ These insights could directly translate to vein grafts, where localized NO delivery at the adventitial surface could simultaneously suppress vascular smooth muscle cell proliferation and support re-endothelialization. Building on this concept, perivascular hydrogels have been engineered. Recent work on hyaluronic acid–dopamine hydrogels has highlighted important considerations for perivascular drug delivery. Traditional crosslinking using sodium periodate generates stiff, brittle gels that adhere well but cannot conform to curved vascular surfaces and raise cytocompatibility concerns, with only ∼59% cell viability reported. To overcome these limitations, a pH-induced crosslinking strategy was developed to avoid oxidative damage, producing softer, more elastic, bio-adhesive hydrogels better suited for application to living vessels. These modified hyaluronic acid–dopamine gels demonstrated improved handling, safer interaction with vascular tissues, and effective drug-release properties, including sustained delivery of the antirestenotic agent atorvastatin, supporting their potential as periadventitial depots for vein graft therapeutics.^267^^,^^268^ Similar perivascular approaches have used Pluronic F-127 gel to deliver small molecules and oligonucleotides, demonstrating consistent inhibition of neointimal thickening in carotid injury and graft models.^269^^,^^270^

Overall, the nonviral delivery literature in vein graft disease and related vascular models supports a clear conclusion: although nonviral systems generally lag behind viral vectors in raw transduction efficiency, they are promising in safety, tenability, and compatibility with intraoperative, perivascular application.

### E CRISPR-based technologies

CRISPR technology has emerged as a highly promising platform for gene therapy in the treatment of many cardiovascular diseases. Initially, CRISPR technology was used as a powerful gene-editing tool in biomedical research, capable of editing mammalian^271^ and human cells,^272^ as well as both small^273^^,^^274^ and large animals,^275^, ^276^, ^277^ with remarkable specificity and efficiency. Today, the versatility of CRISPR has extended beyond conventional genome editing to encompass gene regulation, thereby enabling the development of exciting and innovative next-generation therapeutic strategies. A full description of the CRISPR-Cas system is beyond the scope of this review; readers are referred to recent excellent reviews on this topic.^278^

#### 1 Clinical use of CRISPR-based therapeutics

In November 2023, the field of genome editing achieved a ground-breaking advance when the United Kingdom. Medicines and Healthcare Products Regulatory Agency approved the first and only CRISPR-based therapy so far, CASGEVY.^279^ A few months later, the United States. US Food and Drug Administration and the European Medicines Agency also approved the CRISPR/Cas9 gene therapy. Motivated by these success stories, a growing number of clinical and preclinical investigations have used CRISPR-based genome editing for therapeutic applications across diverse biomedical fields, including cardiovascular disease.^280^ Despite these advances, no clinical or preclinical studies have yet implemented CRISPR technology in the context of vein graft failure. Nevertheless, CRISPR applicability has been studied in vascular pathologies closely associated with vein graft disease, such as atherosclerosis.^281^, ^282^, ^283^, ^284^, ^285^, ^286^

#### 2 CRISPR-based gene regulation: New possibilities

CRISPR has extended beyond genome editing to a wide range of applications. By using a modified, catalytically inactive form of the Cas9 protein known as dCas9 (“dead” Cas9), researchers can precisely target specific genomic regions without introducing double-strand breaks in the DNA and thus, genome editing. This feature allows dCas9 to serve as a flexible platform for attaching various functional proteins, for instance, regulatory molecules that modulate gene expression.

When dCas9 is linked to a transcriptional repressor, it gives rise to a system known as CRISPR interference (CRISPRi). Similar in concept to RNA interference, CRISPRi represses gene expression but operates through a distinct mechanism. Whereas RNA interference relies on nucleotide complementarity and the RNA-induced silencing complex to degrade target mRNA, CRISPRi uses dCas9 fused to a repressor domain to bind a specific DNA sequence and block transcriptional initiation. Conversely, coupling dCas9 to a transcriptional activator such as VP64 or the tripartite VP64-p65-Rta, produces a CRISPR activation (CRISPRa) system capable of enhancing gene expression. Alternatively, fusions with epigenetic regulators such as demethylases and acetylases can induce active chromatin states to drive gene expression.^287^^,^^288^

CRISPR/dCas9 tools have been particularly useful for high-throughput target gene screening^289^^,^^290^; however, the number of cardiovascular research papers using CRISPR/dCas9 systems for therapeutic purposes has increased substantially over the past 5 years. Cardiovascular researchers are using CRISPRa and CRISPRi to reverse genetic dilated cardiomyopathy,^291^^,^^292^ arrhythmogenic cardiomyopathy,^293^^,^^294^ endothelial dysfunction during atherosclerosis^295^^,^^296^ and cardiac trabeculation associated with left ventricular noncompaction.^297^

CRISPRa is particularly appealing because it enables the activation of key transcription factors and cell-identity genes at endogenous levels, offering a safer alternative to methods based on exogenous overexpression. Its reliance on endogenous regulatory mechanisms also allows for the modulation of large, difficult-to-clone genes and complex loci that encompass multiple functional elements, including lncRNA that serve as host genes of miRNAs. As mentioned before, lncRNA have been suggested to possess more cell and tissue-specific expression patterns (153–159), further highlights the potential relevance of these loci to the regulation of cellular identity. In this context, Monteiro et al^298^ demonstrated the activation of the evolutionarily conserved MIR503HG locus in human endothelial cells. MIR503HG encodes 7 lncRNA isoforms and 2 microRNAs, miR503 and miR424, which function together as coordinated regulatory units that preserves endothelial fate and protects against EndMT in vascular disease.^298^ Using a single gRNA targeting shared *cis*-acting regulatory elements, the authors were able to promote the transcription of all components of the locus.^299^ A similar strategy was applied by Vacante et al,^300^ who successfully activated MIR143HG locus in human smooth muscle cells. MIR143HG encodes for the conserved lncRNA CARMN, with more than 50 isoforms, together with miR143 and miR145, forming a well described regulatory unit that promotes the smooth muscle cell contractile phenotype.^301^^,^^302^ CRISPR-mediated activation of the entire locus by targeting the common promoter reversed smooth muscle cell phenotypical switch, representing the first demonstration of a functional effect of CRISPR/dCas9-based therapy in vitro in a context relevant to vascular remodeling and vein graft failure.^300^

**Fig. 6:**
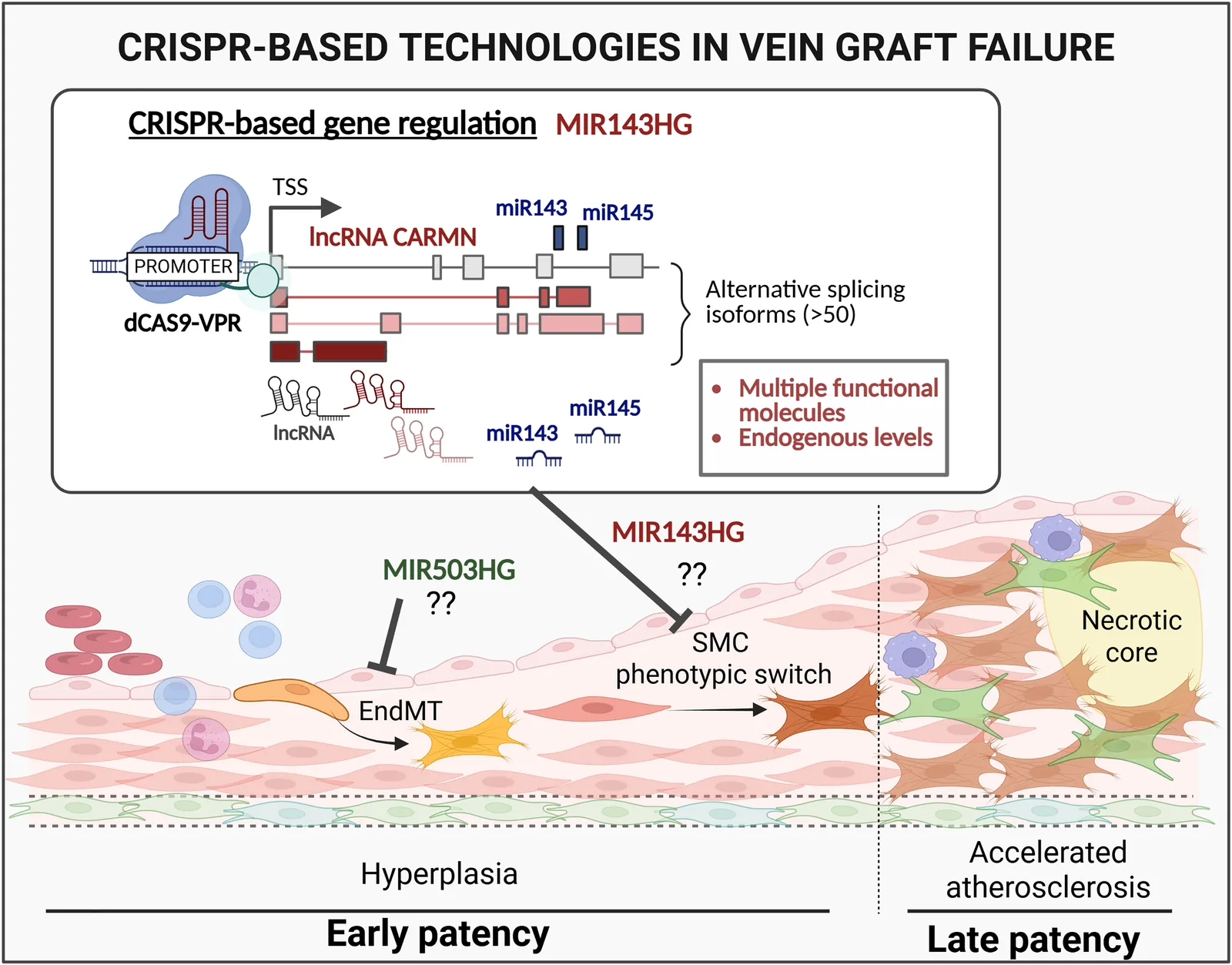
CRISPR-based technologies in vein graft failure. Current knowledge of vein graft failure physiopathology indicates that damage during peri-implantation, together with early exposure to arterial flow, are the main drivers of vein restenosis. The adaptation and early responses of the different vein-cell populations to this damage, including endothelial and smooth muscle cells (SMCs), are critical for long-term vein graft patency. CRISPR-based gene regulation enables the modulation of cell-specific complex loci that are essential for cell fate at endogenous expression levels. Endothelial cell identity is known to be modulated by the MIR503HG locus, which produces 7 lncRNA isoforms and microRNAs miR503 and miR424. The contractile phenotype of SMCs is regulated by the MIR143HG locus, which encodes more than 50 isoforms of lncRNA CARMN, together with miR143 and miR145. Mechanistically, a catalytically inactive dCas9 fused to the tripartite transcriptional activator VP64-p65-Rta (VPR) is guided to the shared promoter region of MIR143HG locus through a specific guide RNA. This recruitment leads to the coordinated expression of all functional molecules derived from MIR143HG locus, thereby preventing the phenotypical switch of SMCs.

In conclusion, CRISPR-based gene regulation is a powerful tool for controlling cell fate by targeting key master regulators at their endogenous levels (Fig. 6). A thorough understanding of the complete transcriptomic landscape in both health and disease will be essential to fully release the potential of these promising approaches and to successfully guide them toward effective clinical therapies for vein graft disease.

### F Advanced biomaterials for venous bypass grafting

The development of artificial scaffolds for CABG and PABG surgery has emerged as a promising response to key limitations of traditional autologous conduits. Early approaches originating in the 1970s used synthetic polymers such as expanded polytetrafluoroethylene and Dacron, which performed well in large-caliber arteries^303^ but failed in small-diameter coronary settings because they are thrombogenic, stiff, and lack the biological cues necessary for re-endothelialization.^304^^,^^305^ These limitations motivated the development of tissue-engineered vascular grafts, designed to mimic the structural, mechanical, and biochemical environment of native vessels. Pioneering in vivo studies at the turn of the 21st century^306^^,^^307^ demonstrated that biodegradable polymer scaffolds could remodel into functional vascular tissue after implantation, and offered excitement at the potential for these to be used in surgical practice. Modern materials science has continued to advance these designs using a variety of approaches. Decellularized scaffolds, typically derived from allogeneic or xenogeneic vessels, preserve the native extracellular matrix architecture while reducing immunogenicity and supporting recellularization.^308^ Although biocompatible and mechanically appropriate, their success depends on successful recellularization, which remains inconsistent.^309^ Biodegradable polymer scaffolds, crafted from materials such as polycaprolactone, polylactic acid, poly(glycolic acid), and their copolymers, aim to provide temporary mechanical support while gradually resorbing as new tissue forms.^310^^,^^311^ Advances in electrospinning, 3D printing, and melt-extrusion techniques have improved control over fiber alignment, pore size, and compliance, enabling scaffolds that more closely replicate coronary artery mechanics.^312^, ^313^, ^314^ Functionalization of scaffolds has also been explored through the incorporation of bioactive molecules such as heparin, VEGF, or nitric-oxide releasing compounds to promote re-endothelialization and reduce thrombosis during the critical early postimplantation phase.^315^, ^316^, ^317^ Finally, hybrid scaffolds combine extracellular matrix components with synthetic backbones emerged from data showing improved cell adhesion and mechanical robustness compared with single-material designs.^318^

A major focus of current development is achieving physiologic compliance, as mismatch between graft and artery can induce disturbed flow and early graft failure.^305^^,^^319^ Significant research now uses computational modeling,^320^ mechanobiology-driven design,^321^ and dynamic bioreactor conditioning^322^ toward improved scaffold design and optimization, which aims to conditioning grafts to withstand coronary pulsatility and shear stress. Despite substantial progress, challenges remain, including efficient and complete re-endothelialization, prevention of thrombosis without prolonged anticoagulation, long-term resistance to atherosclerosis, and reliable large-scale manufacturing are among the most common setbacks. Despite these, engineered grafts have entered, and indeed, completed clinical trials with encouraging safety outcomes but limited long-term data.^323^, ^324^, ^325^ The integration of advanced polymers, regenerative biology, and precision fabrication technologies suggests that synthetic scaffolds may soon offer viable alternatives for patients lacking suitable autologous vessels, with the potential to provide physiologically compliant, endothelialized conduits capable of long-term remodeling.

Photochemical tissue passivation is a technique that uses a photosensitizing dye (such as Rose Bengal dye) coated on the adventitial layer of the vessel, which when excited using LED light, crosslinks collagen to the extracellular matrix in the adventitial layer of the blood vessel, effectively stiffening the vessel before grafting.^326^ Ex vivo studies have demonstrated in both rat^326^ and pig^327^ vein graft models reduced intimal area and reduced smooth muscle cell proliferation in photochemical tissue passivation-treated tissues. This is being explored commercially by DurVena in a phase 1 clinical trial (NCT06150872); however, no data have been published to date.

## V Conclusions

SVG failure after CABG and PABG is a global problem with significant setbacks to patients and healthcare systems. Research continues to define key pathways and processes that underpin this complex disease, whereas advancements in single cell genomics technologies are illuminating how widespread changes in cell heterogeneity develop during the remodeling process. These areas, coupled with extensive advances in gene and RNA therapeutics and advanced imaging techniques offer hope of new, targeted therapeutic approaches to prevent occlusive vein graft disease.

## Conflict of interest

Andrew H. Baker has patents granted in European Union (EU; validated in United Kingdom, Germany, and France) (EP3271459) and United States (US10588919, 19/104460); EU (23761973.9); China (202380060569.4); and Japan (2025508959) related to compounds and targets discussed in this review. Andrew H. Baker and Stuart A. Nicklin have a patent granted on HAdV-49 (patents filed by Crucell Holland BV to transfer jointly to University of Glasgow/Edinburgh). Colin Berry is employed by the University of Glasgow, which holds consultancy and research agreements for his work with Abbott Vascular, AskBio, AstraZeneca, Boehringer Ingelheim, CorFlow, Edwards Lifesciences, MAIA Pharmaceuticals, Merck, Servier, Novartis, and Vahaticor. All other authors declare no conflicts of interest.
